# Tubulin detyrosination shapes *Leishmania* cytoskeletal architecture and virulence

**DOI:** 10.1073/pnas.2415296122

**Published:** 2025-01-14

**Authors:** Rosa Milagros Corrales, Jeremy Vincent, Lucien Crobu, Rachel Neish, Binita Nepal, Julien Espeut, Grégoire Pasquier, Ghislain Gillard, Chantal Cazevieille, Jeremy C. Mottram, Dawn M. Wetzel, Yvon Sterkers, Krzysztof Rogowski, Maude F. Lévêque

**Affiliations:** ^a^Maladies infectieuses et Vecteurs: Ecologie, Génétique, Evolution et Contrôle, University of Montpellier, CNRS, Institut de Recherche pour le Développement, Montpellier 34095, France; ^b^York Biomedical Research Institute, Department of Biology, University of York, York YO10 5DD, United Kingdom; ^c^Department of Pediatrics, University of Texas Southwestern Medical Center, Dallas, TX 75390; ^d^Department of Biochemistry, University of Texas Southwestern Medical Center, Dallas, TX 75390; ^e^Tubulin Code team, Institute of Human Genetics, CNRS, Université Montpellier, Montpellier 34090, France; ^f^Université Montpellier, INSERM U1298, Institute for Neurosciences of Montpellier, Montpellier 34090, France

**Keywords:** trypanosomatids, microtubules, vasohibin, dynamics, kinesin-13

## Abstract

Tubulin detyrosination has been implicated in various human disorders and is important for regulating microtubule dynamics. While in most organisms this modification is restricted to α-tubulin, in trypanosomatid parasites, it occurs on both α- and β-tubulin. Here, we show that in *Leishmania*, a single vasohibin (LmVASH) enzyme is responsible for differential kinetics of α- and β-tubulin detyrosination. LmVASH knockout parasites, which are completely devoid of detyrosination, show decreased levels of glutamylation and exhibit a strongly diminished pathogenicity in mice, correlating with decreased proliferation in macrophages. Reduced virulence is associated with altered morphogenesis and flagellum remodeling in detyrosination-deficient amastigotes. Flagellum shortening in the absence of detyrosination is caused by hyperactivity of a microtubule-depolymerizing Kinesin-13 homolog, demonstrating its function as a key reader of the trypanosomatid-tubulin code. Taken together, our work establishes the importance of tubulin detyrosination in remodeling the microtubule-based cytoskeleton required for efficient proliferation in the mammalian host. This highlights tubulin detyrosination as a potential target for therapeutic action against leishmaniasis.

## Significance

Leishmaniasis, the most lethal parasitic disease after malaria, represents a major threat to global health. *Leishmania* parasites undergo extensive morphogenesis during their life cycle, relying on a highly adaptable microtubule-based cytoskeleton. Although tubulin posttranslational modifications exist in *Leishmania,* their role in cytoskeletal regulation remains poorly understood. Here, we show the pivotal role of tubulin detyrosination in *Leishmania* pathogenicity. By identifying LmVASH as the sole enzyme responsible for differential detyrosination of α- and β-tubulin, we reveal its impact on microtubule dynamics and cytoskeletal architecture. The lack of detyrosination results in a strongly reduced proliferation within infected macrophages and dramatically diminished pathogenicity in mice. This study establishes tubulin detyrosination as crucial for cytoskeleton remodeling and as a promising target for therapeutic intervention.

Microtubules are highly dynamic polymers formed by the assembly of α- and β-tubulin heterodimers. They stochastically switch between phases of growth and shrinkage, called dynamic instability (1), and fulfill a wide variety of important cellular functions ranging from cell division, maintaining the cell shape to intracellular transport and cell motility. Each specific function requires the recruitment of a defined set of microtubule-interacting proteins, many of which bind to the C-terminal tails of both tubulins that are present on the microtubule surface. The variability of tubulin tails is provided either by the expression of different α- and β-tubulin isotypes or through posttranslational modifications (PTMs), including the addition or removal of amino acids. The combination of these two mechanisms, collectively referred to as the “tubulin code,” generates locally restricted patterns on microtubules, which are recognized by a specific subset of microtubule-associated proteins (MAPs) and molecular motors (2). The recruited proteins can in turn regulate microtubule dynamics either by stabilizing or destabilizing them, direct motor-dependent transport, or connect microtubules to other cellular structures (3).

The first tubulin modification to be discovered was detyrosination, which consists in the removal of the very C-terminal tyrosine from α-tubulin leading to the formation of the so-called αΔ1-tubulin. The importance of detyrosination in human health and disease has already been well established. It serves as a readable code for the specific recruitment of MAPs, regulating various biological processes such as mitosis, neuronal transport, cardiomyocyte contraction as well as myogenesis and neurogenesis (2). Detyrosinated microtubules have been shown to confer preferential binding for specific motors, allowing a modification-dependent loading of selected cargos and microtubule modulators (4, 5). On the other hand, tyrosinated microtubules regulate dynein-dependent motility (6) and drive the binding of Cap-Gly domain-containing proteins controlling microtubule growth speed and longevity (7). Whereas detyrosinated α-tubulin is a marker of long-lived microtubules, dynamic microtubules are mainly composed of tyrosinated α-tubulin. A mechanistic explanation to correlate microtubule stability to α-tubulin detyrosination is supported by increased activity of depolymerizing enzymes from the Kinesin-13 family toward tyrosinated microtubules (5, 8).

Although detyrosination has been known for almost half a century, the modifying enzymes have been discovered only recently. The first type of detyrosinating enzymes was shown to be the two members of the Vasohibin family, VASH1 and VASH2 (9, 10). More recently, two independent studies identified a second class of detyrosinases named either Microtubule Associated Tyrosine Carboxypeptidase (11) or Tubulin MetalloCarboxyPeptidase 1 (12), both corresponding to the same gene. The reverse reaction is catalyzed by a Tubulin Tyrosine Ligase (TTL), which adds a tyrosine back to the terminal site of detyrosinated α-tubulin (13).

Tubulin detyrosination is highly evolutionarily conserved being present in organisms that diverged from the main eukaryotic lineage such as trypanosomatids (14) that include: *Leishmania* spp., *Trypanosoma cruzi,* and *Trypanosoma brucei,* responsible for leishmaniasis, Chagas disease, and African trypanosomiasis, respectively. They are collectively known as the TriTryps and have been identified as a major public health problem and targeted by the WHO road map for neglected tropical diseases (15). All three parasites have complex life cycles alternating between a mammalian host and an insect vector. This implies different developmental stages that rely on a highly adaptable cytoskeleton to accommodate major changes in the parasite’s morphology. Although all these parasites undergo complex life cycle, their cytoskeleton relies almost completely on microtubule-based structures including i) the subpellicular corset, a helical single layer of highly ordered MTs that defines the parasite’s shape, ii) the intranuclear mitotic spindle which is assembled during a closed mitosis and iii) the single flagellum essential for cellular motility and infectivity (16). Another specialized microtubule-based assembly is a set of four microtubules known as the microtubule quartet (MtQ) that nucleates close to the basal bodies and wraps around the flagellar pocket membrane (17, 18). *Leishmania* have an additional set of microtubules associated with the tubular lysosome which runs along the anterior–posterior axis of the cell and dynamically changes through the cell cycle (19).

Trypanosomatids express a limited number of α- and β-tubulin isotypes making PTMs a potential key factor for microtubule specialization. While *T. brucei* and *T. cruzi* express only a single α- and β-tubulin isotype, the *Leishmania* genome contains one additional β-tubulin gene (16), which is differentially regulated during the life cycle (20). Hence, the variable primary sequence and expression of *Leishmania* β-tubulin genes may provide additional regulatory mechanisms to the prototype tubulin code of trypanosomatid parasites. Furthermore, while detyrosination in other eukaryotes is restricted to α-tubulin, in trypanosomatids it occurs on both α- and β-tubulin (21) providing an additional layer of complexity to their “tubulin code.”

The presence of this noncanonical C-terminal tyrosine on β-tubulin may play an important role in a particular developmental stage of these medically important pathogens. In contrast to mammals, which express several detyrosinating enzymes (9–12), we have previously shown that detyrosination in *T. brucei* is catalyzed by a single Vasohibin (VASH) homolog which modifies both α and β-tubulin subunits (22). Furthermore, we demonstrated that the loss of this modification has important consequences for cell cycle progression and maintenance of procyclics (insect form) morphology (22). Detyrosination, however, remains unexplored in other trypanosomatids such as *Leishmania*, a parasite responsible for 700,000 to 1 million new leishmaniasis cases annually (23).

The *Leishmania* life cycle involves two developmental stages. While promastigotes are motile elongated cells displaying extensive morphological changes within the insect vector, the amastigotes which proliferate in mammalian macrophages are characterized by a spherical shape with a short immotile flagellum. The transition between the two forms involves complex morphological changes, which require a highly adaptable microtubule-based cytoskeleton and as such might be regulated by tubulin detyrosination. To address the function of this modification during the parasite’s morphogenesis, we used *Leishmania* parasites that can be readily converted from promastigotes to amastigotes.

Here, we describe a comprehensive analysis of tubulin detyrosination in *Leishmania.* We show that the removal of the C-terminal tyrosine from both α- and β-tubulin is catalyzed by a single VASH homolog, which however shows a preference toward α-tubulin. The lack of detyrosination has important consequences during the *Leishmania* life cycle by affecting microtubule dynamics and cytoskeleton remodeling during the transition from promastigotes to amastigotes. Furthermore, we provide evidence that this modification influences the activity of specific MAPs including a flagellar-resident microtubule depolymerizing enzyme from the Kinesin-13 family. Strikingly, we show that VASH-knockout amastigotes are characterized by a poor proliferation in macrophages and a strongly reduced pathogenicity in mice. These results demonstrate the importance of detyrosination in *Leishmania* virulence and establish LmVASH as an important target for medical intervention.

## Results

### The *Leishmania* VASH Is an Autonomous Tubulin Detyrosinase.

In mammalian cells, the two members of the vasohibin family (VASH1 and VASH2) form a complex with their cofactor, a small vasohibin-binding protein (SVBP), to catalyze the removal of the C-terminal tyrosine from α-tubulin (9, 10). Alike in *T. brucei* (22), no SVBP homolog has been found in the *Leishmania* genome. Moreover, the single homolog of vasohibins in *T. brucei* is an autonomous detyrosinase which is evolutionarily more closely related to VASH2 than to VASH1 (22). Alignment of the amino acid sequences of the predicted *Leishmania mexicana (*LmVASH) vasohibin with its human (HsVASH2) and *T. brucei* (TbVASH) counterparts showed a high degree of conservation within the catalytic domain (31% and 58% identity, respectively) (*SI Appendix*, Fig. S1*A*). Importantly, it contained the catalytic triad residues, including the essential cysteine, which is absolutely required for the enzymatic activity. In contrast to the catalytic domain, the C-terminal extension, which was significantly longer in LmVASH, showed a rather poor homology with HsVASH2 or TbVASH. In addition to the presence of a putative nuclear localization signal (*SI Appendix*, Fig. S1 *A* and *B*), the C-terminal extension may contain specific motifs required either for the interaction with regulatory proteins or for targeting the enzyme to distinct microtubule-based structures.

To determine whether LmVASH has detyrosinase activity, we expressed either a wild-type or a catalytically dead (C92A) version of the enzyme in HEK293 cells. Immunoblot analysis showed a strong increase in α-tubulin detyrosination (α∆1), which was restricted to protein extracts derived from cells expressing the active LmVASH (*SI Appendix*, Fig. S1*C*). These results were further validated by immunofluorescence analysis, which demonstrated an increase in α∆1 labeling specifically in the cells expressing the active but not catalytically dead version of LmVASH (*SI Appendix*, Fig. S1*D*). To confirm that LmVASH directly modifies α-tubulin, we performed in vitro detyrosination assays using recombinant wild-type or catalytically dead (C92A) LmVASH (*SI Appendix*, Fig. S1*E*) and tubulin purified from insect *Spodoptera frugiperda*-derived Sf9 cells (*SI Appendix*, Fig. S1*F*) known to be fully tyrosinated (22). The active enzyme generated α∆1-tubulin signal with concomitant reduction in tyrosination levels, whereas the C92A mutant showed no activity (*SI Appendix*, Fig. S1*G*). Moreover, we found that LmVASH specifically detyrosinated microtubules, while it showed no significant activity toward free tubulin dimers (*SI Appendix*, Fig. S1 *H* and *I*). Taken together our results demonstrate that LmVASH is an autonomously active tubulin detyrosinase.

### Distinct Distribution of α- and β-Tubulin Detyrosination in *Leishmania*.

**Fig. 1.**
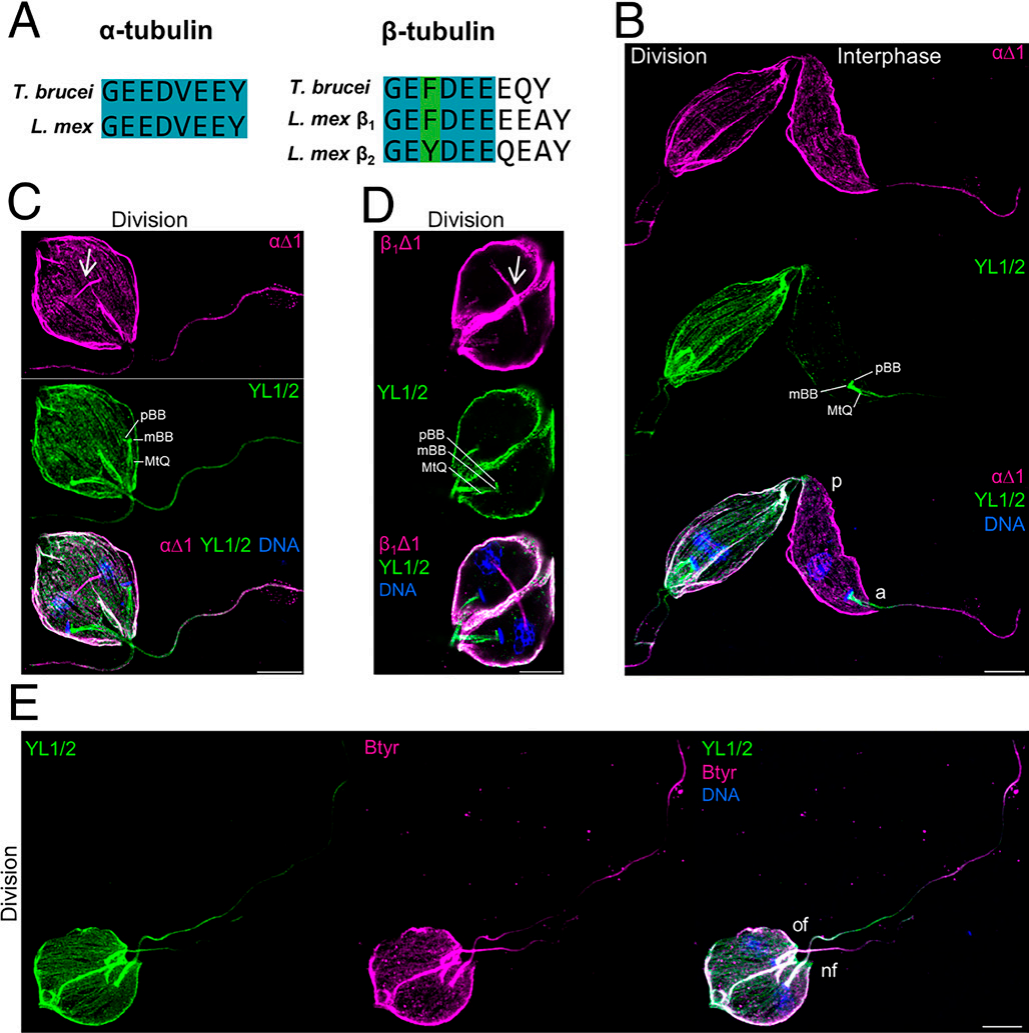
Distinct distribution of α- and β-tubulin detyrosination in *Leishmania*. (*A*) Sequence alignment of tubulin tails. *T. brucei* α-tubulin isotype located on chromosome 1, *L. mexicana* α-tubulin isotype located on chromosome 13, *T. brucei* β-tubulin isotype located on chromosome 1, *L. mexicana* β_1_-tubulin isotype located on chromosome 8 and 32 and *L. mexicana* β_2_-tubulin isotype located on chromosome 21. Blue and green colors correspond to conserved and equivalent residues, respectively. (*B*–*E*) UExM analysis of tyrosinated and detyrosinated α and β-tubulin in *L. mexicana* parental promastigotes. Maximum intensity projections (*B*, *C*, and *E*) and a single section (*D*) of z-stack confocal images are presented. The αΔ1 antibody recognizes detyrosinated α-tubulin. The YL1/2 antibody recognizes tyrosinated α-tubulin. The β_1_Δ1 antibody recognizes detyrosinated β-tubulin. The βtyr antibody recognizes tyrosinated β-tubulin. (*C* and *D*) White arrows indicate the mitotic spindle. p: posterior;.a: anterior; of: old flagellum; nf: new flagellum; MtQ: Microtubule quartet; mBB: mature basal body: pBB: pro-basal body. Nb: only one MtQ, mBB, and pBB is highlighted in each dividing cells. (Scale bar: 10 µm.)

Since the sequence of the C-terminal tail of *Leishmania* α-tubulin is identical to that of *T. brucei* (Fig. 1*A*), we took advantage of already available antibodies including YL1/2 and Tb α∆1 to analyze the distribution of tyrosinated and detyrosinated α-tubulin respectively (22). This allowed us to identify a differential tyrosination/detyrosination pattern of distinct microtubule structures during *Leishmania* cell cycle. In interphasic cells, while most subpellicular microtubules were found to be labeled by α∆1 antibody (Fig. 1*B* and Movie S1), the growing posterior region of the cell contained exclusively tyrosinated microtubules (*SI Appendix*, Fig. S2 *A* and *B*). In contrast to constitutive tyrosination of the flagellum tip in *T. brucei* interphasic cells (24), the growing end of *L. mexicana* flagella only occasionally contained tyrosinated α-tubulin (*SI Appendix*, Fig. S2*A*). The anterior region of the cell body was stained by the YL1/2 antibody (*SI Appendix*, Fig. S2*A*), which labeled the pro-basal and basal bodies, the internalized flagellum as well as the MtQ (Fig. 1*B* and Movie S2). All three microtubule assemblies remained constitutively tyrosinated throughout the cell cycle, while detyrosinated α-tubulin was exclusively detected in flagella that extended outside of the cell body (Fig. 1 *B* and *C* and Movies S1 and S2). This pattern is consistent with the flagellar localization of an episomal GFP-fused LmVASH (*SI Appendix*, Fig. S2*C*) and suggests that LmVASH-mediated detyrosination occurs within the externalized flagellum. In dividing cells, the growing end of the two external flagella contained mostly tyrosinated tubulin (*SI Appendix*, Fig. S2*E*). Moreover, the overall level of tyrosinated α-tubulin was found to be increased in the subpellicular microtubules (Fig. 1*B* and Movie S1). These results could be consistent with the newly assembled, and thus tyrosinated, microtubules being intercalated into the preexisting detyrosinated subpellicular corset as previously observed in *T. brucei* (25). In addition, spindle microtubules were found to contain exclusively detyrosinated α-tubulin as shown by conventional (*SI Appendix*, Fig. S2*E*) and expansion (Fig. 1*C* and Movie S2) microscopy. This suggests that the modification of the spindle microtubules takes place in the nucleus, which is consistent with the nuclear localization of episomal GFP-LmVASH (*SI Appendix*, Fig. S2*C*) and endogenously HA-tagged LmVASH (*SI Appendix*, Fig. S2*D*) present on the mitotic spindle. Alike in *T. brucei* (22), LmVASH was not detected at the cortical microtubules (*SI Appendix*, Fig. S2 *C* and *D*). This may reflect low expression levels, transient localization, and/or high turnover rate on this specific microtubule compartment.

In trypanosomatids, apart from α-tubulin, β-tubulin also carries the very C-terminal tyrosine and as such might be subjected to detyrosination. In contrast to *T. brucei*, *Leishmania* encodes two different isotypes of β-tubulin, β_1_ and β_2_, present on three distinct chromosomes (26) (Fig. 1*A*). To test whether detyrosination also occurs on β-tubulin, we raised *L. mexicana*-specific antibodies for tyrosinated (βtyr) and detyrosinated (β∆1) β-tubulins using the C-terminal sequence of the most abundant β_1_ isotype. The β∆1 antibody positively labeled mitotic spindles (Fig. 1*D*, *SI Appendix*, Fig. S2*F*, and Movie S3), however, the signal was mostly detected using expansion microscopy, while conventional immunofluorescence typically yielded negative staining. This pattern suggests higher activity of LmVASH toward α-tubulin as compared to β-tubulin. To investigate this observation further, we compared the tyrosination pattern of α- and β-tubulin in the interphasic (*SI Appendix*, Fig. S2*G* and Movie S4) and dividing (Fig. 1*E* and Movie S5) promastigotes by labeling them with YL1/2 and βtyr antibodies. We found that in interphasic cells the growing posterior end and the anterior region of the cell body, which includes the pro-basal and basal body as well as internalized flagellum and the MtQ, showed overlapping signals (*SI Appendix*, Fig. S2*G* and Movie S4). Such pattern, combined with the lack of labeling of the anterior compartments by both α∆1 (Fig. 1 *B* and *C* and Movies S1 and S2) and β∆1 (Fig. 1*D* and Movie S3) antibodies, indicates that these microtubule structures are not subjected to the modification by LmVASH. By contrast, the labeling of the old external flagellum was found to be heterogeneous, revealing differential tyrosination patterns of α- and β- tubulin along the old flagellum in dividing cells (Fig. 1*E* and Movie S5). While the proximal portion of the externalized flagellum was found to contain mainly tyrosinated α-tubulin, the distal part was enriched in tyrosinated β-tubulin. Such pattern further suggests that the two tubulin subunits are modified with different kinetics by LmVASH. Overall, the resolution provided by expansion microscopy revealed heterogeneous distribution of α- and β-tubulin detyrosination in distinct microtubule structures during the *Leishmania* cell cycle.

### Deletion of LmVASH Reveals Differential Kinetics of Detyrosination and a Cross-Talk with Polyglutamylation.

**Fig. 2.**
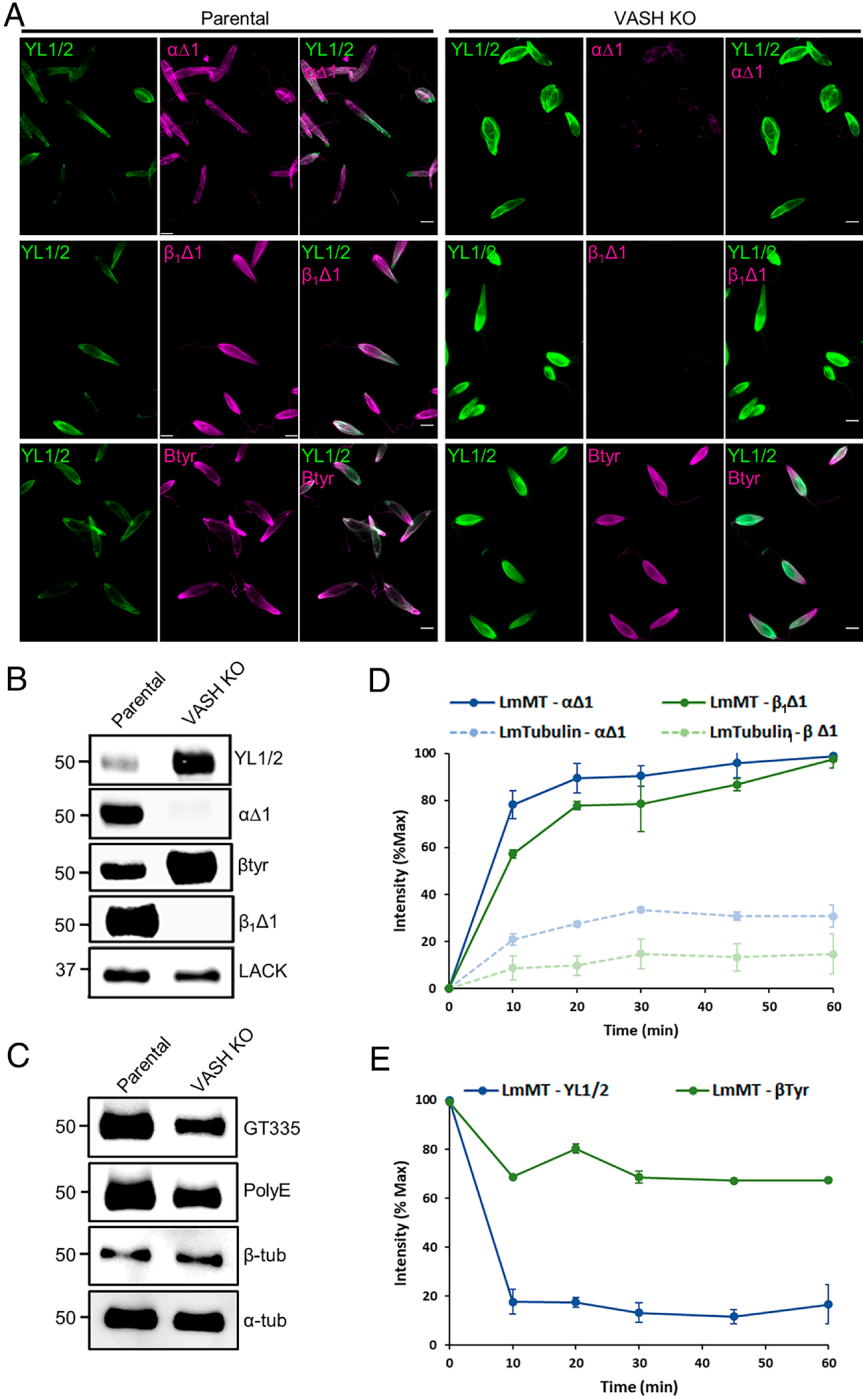
Deletion of LmVASH reveals differential kinetics of detyrosination and a cross-talk with polyglutamylation. (*A*) Immunofluorescence and (*B*) immunoblot analysis of tyrosinated and detyrosinated α and β-tubulin in cytoskeletons from *L. mexicana* parental and LmVASH knockout promastigotes. Note the absence of α and β-tubulin detyrosination and increase levels of α and β-tubulin tyrosination signal in LmVASH knockout cells. LACK is used as a loading control. (Scale bar: 5 µm.) (*C*) Immunoblot analysis of tubulin PTMs in cytoskeletons from *L. mexicana* parental and LmVASH knockout promastigotes. In the absence of detyrosination, LmVASH knockout cells showed decreased levels of polyglutamylation. The GT335 antibody recognizes glutamate side chains of any length. The PolyE antibody recognizes polyglutamylation with at least four glutamate residues. α-tubulin is used as a loading control. (*D* and *E*) In vitro time course analysis of the recombinant wild-type LmVASH-mediated α- and β- tubulin detyrosination. Samples were analyzed by immunoblotting (*SI Appendix*, Fig. S3*F*) and relative optical density of three independent assays was measured (n = 3; error bars represent SEM). (*D*) Comparison of detyrosination activity on polymerized (MT) and free tubulin purified from LmVASH knockout promastigotes was performed based on relative optical densities generated by the αΔ1 and the β_1_Δ1 antibodies. Note that LmVASH enzyme displays detyrosinase activity toward soluble and polymerized *Leishmania* tubulin. (*E*) Comparison of detyrosination activity on polymerized tubulin (MT) from LmVASH knockout promastigotes was performed based on relative optical densities generated by the YL1/2 and the βtyr antibodies. Note that LmVASH enzyme displays slower kinetics of β-tubulin detyrosination toward polymerized *Leishmania* tubulin. The αΔ1 antibody recognizes detyrosinated α-tubulin. The YL1/2 antibody recognizes tyrosinated α-tubulin. The β_1_Δ1 antibody recognizes detyrosinated β-tubulin. The βtyr antibody recognizes tyrosinated β-tubulin.

To address the functional importance of LmVASH, we generated null mutant parasites using the CRISPR-Cas9 system (27). The integration of the resistance markers and loss of the LmVASH open reading frame were confirmed by PCR (*SI Appendix*, Fig. S3*A*). Immunofluorescence analysis of the knockout (KO) parasites revealed a complete loss of the staining for detyrosinated α- and β-tubulin as compared to parental cells, indicating that LmVASH catalyzes the modification of both tubulin subunits (Fig. 2*A*). Moreover, we observed increased labeling of tyrosinated tubulin, which became evenly distributed throughout the cell (Fig. 2*A*). In addition, the exclusive labeling of mitotic spindles by YL1/2 but not detyrosination-specific antibody in the LmVASH KO cells further confirmed that the modification of spindle microtubules by LmVASH occurs in the nucleus (*SI Appendix*, Fig. S3*B*). These findings were further confirmed by immunoblot analysis of cell lysates, which showed a complete absence of α- and β-tubulin detyrosination in LmVASH KO cells with simultaneous increase in the levels of tyrosinated signal for both tubulins (Fig. 2*B*). This result demonstrates that LmVASH modifies both α- and β-tubulin subunits and that it is the only tubulin detyrosinase present in *Leishmania*.

Considering that tubulin detyrosination was shown to be associated with stable microtubules, we next examined the status of other tubulin PTMs including acetylation and polyglutamylation. While tubulin acetylation was not affected by the absence of detyrosination (*SI Appendix*, Fig. S3*C*), a strong decrease in the level of tubulin polyglutamylation was observed as demonstrated by two different antibodies: GT335, which recognizes the branching point glutamate, and PolyE, which is specific to long glutamate chains composed of at least four glutamates (Fig. 2*C*). Similar reduction in the level of polyglutamylation was also detected in extracts from *T. brucei* VASH-KO cells as compared to the parental strain (*SI Appendix*, Fig. S3*D*). Altogether, these results demonstrate that tubulin detyrosination positively regulates polyglutamylation.

Next, to assess the enzymatic preference of LmVASH either toward the α- or β-tubulin, we set up an in vitro detyrosination assay using recombinant LmVASH enzymes and fully tyrosinated tubulin purified from the LmVASH-KO cell line. The tubulin from KO promastigotes was used as an unmodified substrate and purified as previously described for *L. tarentolae* (28) (*SI Appendix*, Fig. S3*E*). The wild-type enzyme modified both α- and β-tubulin, whereas the catalytically inactive LmVASH (C92A) did not modify either subunit (*SI Appendix*, Fig. S3*F*). We further investigated LmVASH kinetics toward each tyrosinated subunit using in vitro time course analysis of detyrosinase activities using either soluble tubulin or microtubules incubated with the wild-type enzyme. This assay is based on quantifying signal intensity from western blots using antibodies specific to tyrosinated or detyrosinated α- or β-tubulin (*SI Appendix*, Fig. S3*G*), allowing us to monitor tyrosination changes over time for each subunit. Unexpectedly, while LmVASH activity on insect α-tubulin was restricted to microtubules (*SI Appendix*, Fig. S1 *H* and *I*), the enzyme displayed autonomous detyrosinase activity toward soluble *Leishmania* tubulin in addition to its strong activity on microtubules (Fig. 2*D* and *SI Appendix*, Fig. S3*G*). Moreover, we observed a faster decline in the signal generated by the antibody specific to tyrosinated α-tubulin (YL1/2) compared to the signal from the antibody specific to tyrosinated β-tubulin (β-tyr), whether tubulin was in polymerized form (Fig. 2*E* and *SI Appendix*, Fig. S3*G*) or soluble (*SI Appendix*, Fig. S3 *G* and *H*). This shows that LmVASH preferentially modifies α-tubulin subunit regardless of their polymerization status. Taken together, these results indicate that α- and β-tubulin are differentially modified and that detyrosination acts as a positive regulator of polyglutamylation, thus revealing an unexpected crosstalk between the two modifications.

### Removal of LmVASH Carboxypeptidase Activity Reduces Replication within Macrophages and Pathogenicity in Mice.

In *Leishmania*, the transition from promastigotes to amastigotes involves complex morphological changes that require a highly adaptable microtubule-based cytoskeleton, potentially regulated by tubulin detyrosination.

**Fig. 3.**
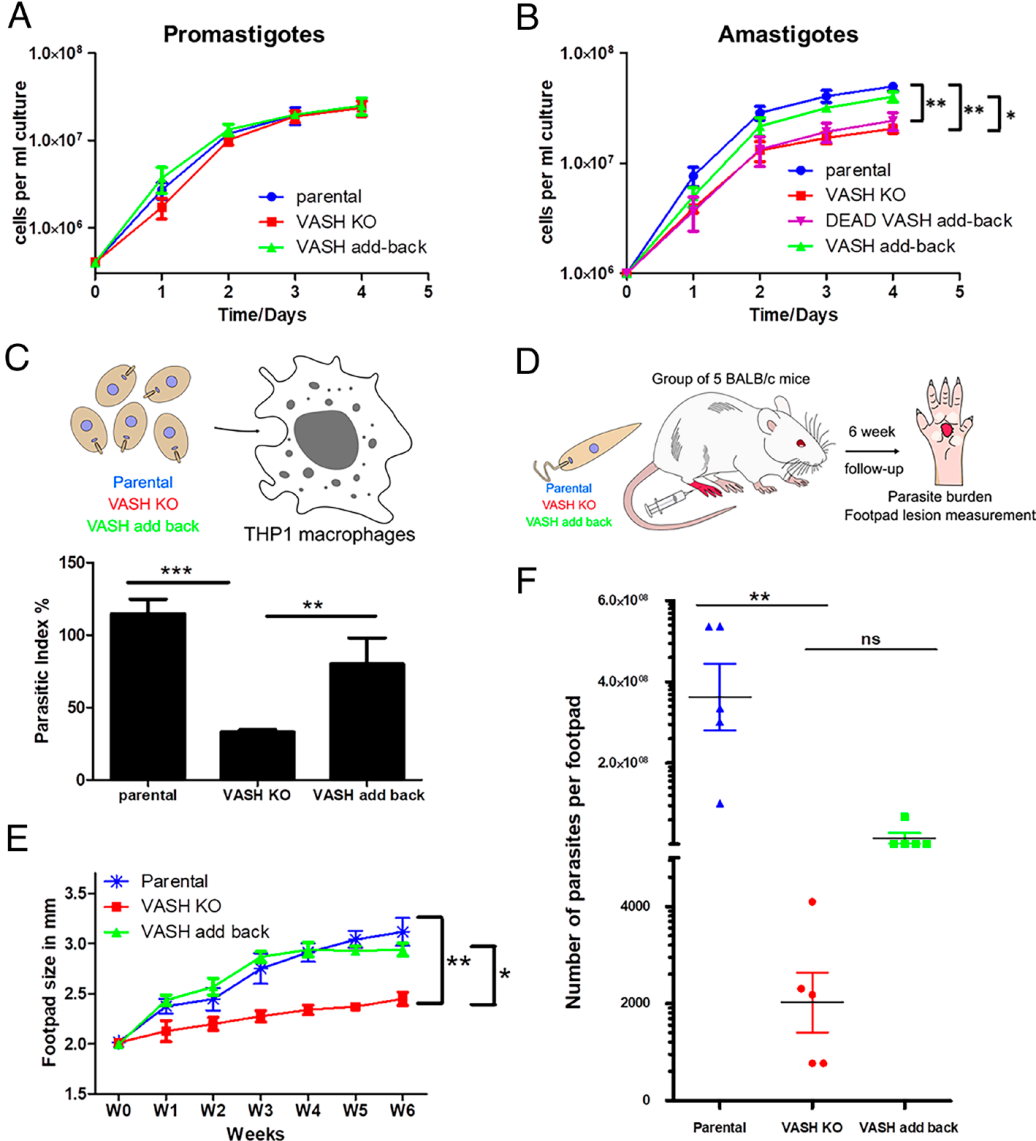
Removal of LmVASH carboxypeptidase activity reduces replication within macrophages and pathogenicity in mice. (*A*) Representative growth curve (log scale) of *L. mexicana* promastigotes cultured over 4 d from parental, LmVASH knockout and wild-type LmVASH add-back cell lines. Cell density was determined by counting at 24 h intervals (n = 3; error bars represent SD). (*B*) Growth curve (log scale) of *L. mexicana* axenic amastigotes obtained after 3 d of differentiation and cultured over a 4-d course from parental, LmVASH knockout, wild-type and enzymatically dead LmVASH complemented cell lines. Cell density was determined by counting at 24 h intervals (n = 3; error bars represent SD). The *P* value was calculated using two-tailed unpaired Student’s *t* test comparing each cell line at day 4. **P < 0.01, *P < 0.05. (*C*) Schematic representation of THP-1 macrophage infection. Axenic amastigotes of parental, LmVASH knockout and LmVASH add-back cells were used to infect differentiated THP-1 monocytes. Note that deletion of LmVASH leads to reduction of the parasitic index (PI) in infected THP-1 cells. The PI was defined as the percentage of infected macrophages x number of intracellular parasites/macrophage. ***P < 0.001, **P < 0.01. (*D*) Schematic representation of the experimental mouse model used to analyze *Leishmania* virulence in vivo. Mice footpads were infected either with the parental, null mutant or add-back stationary phase promastigotes and the infection progress was evaluated over a 6-wk time course. (*E*) Measurement of mean footpad lesion size during a 6-wk infection time course showing that infection with LmVASH knockout produces smaller lesions compared to parental and LmVASH add-back stationary-phase promastigotes. Error bars represent SDs. The *P* value was calculated using two-tailed unpaired Student’s *t* test comparing each cell line at week 6. **P < 0.01, *P < 0.05. (*F*) Measurement of parasite burden at the end of the 6-wk infection time course in the footpad lesion showing that infection with LmVASH knockout leads to a significant drop of parasite burden compared to the parental and LmVASH add-back cells. The parasite number from each infection is plotted, with the mean and the 95% SEM interval indicated. **P < 0.01 (Student’s *t* test).

Initially, we focused our analysis on promastigotes by evaluating the growth rate of parental, LmVASH KO cells and rescued KO parasites expressing episomal HA-LmVASH. We found no significant difference among the three strains (Fig. 3*A*). Given that detyrosination in the promastigote accumulates on externalized flagellum (Fig. 1*B*), we performed motility assays to evaluate the effect of tubulin tyrosination on swimming speed and directionality (velocity/speed) as previously described (29). Knockout cells showed no differences in the average swimming speed as compared to the parental or rescue cell lines (*SI Appendix*, Fig. S4*A*). However, the average swimming directionality of the KO cells was significantly reduced (*SI Appendix*, Fig. S4*B*).

Next, we induced differentiation of promastigotes into amastigotes, a transition that can be triggered in axenic cultures with environmental cues of pH and temperature (30). We monitored cellular growth after 3 d of differentiation and observed reduced proliferation of LmVASH-deleted amastigotes, which was rescued by episomal complementation with the wildtype but not catalytically dead version of LmVASH (Fig. 3*B*). These results clearly show that detyrosinase activity of LmVASH is required for the proper proliferation of amastigotes (Fig. 3*B*). Immunoblot analysis involving the protein extracts from differentiated cells consistently showed complete loss of α- and β-tubulin detyrosination with concomitant increased in tyrosination levels in the amastigotes lacking LmVASH (*SI Appendix*, Fig. S4*C*). Likewise, as observed in promastigotes, deletion of LmVASH in amastigotes resulted in complete lack of detyrosination on mitotic spindles (*SI Appendix*, Fig. S4*D*) and reduced levels of glutamylation with no impact on the level of acetylation (*SI Appendix*, Fig. S4*E*).

In *Leishmania*, the formation of a new flagellum (F), the division of the kinetoplast (K) (mitochondrial DNA) and the nucleus (N) are tightly orchestrated during the cell cycle. The cell cycle stage may thus be schematically defined by the number of flagella, kinetoplasts, and nuclei. To assess whether cell cycle defects might be responsible for the reduced growth of the KO parasites, we stained the parental and KO cell lines expressing a flagellar membrane marker SMP1-mCherry with Hoechst and determined the N/K/F patterns in the axenic amastigote stage (*SI Appendix*, Fig. S4*F*). The KO cell line showed a reduced number of interphasic (1N1K1F) cells and an increase in abnormal cells displaying odd morphotypes (0N0K0F, 0N1K1F, and 1N0K0F) (*SI Appendix*, Fig. S4*F*) suggesting defects in cell division. To further confirm this observation, we analyzed the chromosomal contents of interphasic amastigotes using a fluorescence in situ hybridization probe specific to chromosome 2 (31). In agreement with the remarkable genomic plasticity of *Leishmania* parasites (32), the parental population displayed both monosomy and disomy for this chromosome (*SI Appendix*, Fig. S4*G*). In the KO population, the proportion of disomic cells was significantly reduced and correlated with a slight increase in monosomic cells suggesting an abnormal chromosomal distribution in the absence of detyrosination. In summary, these results showed that deletion of LmVASH hinders motility without preventing cell proliferation in the insect form while detyrosinase activity of LmVASH is required for proper cell division in the mammalian form.

To establish a successful infection, *Leishmania* parasites must enter and multiply inside macrophages. Since the lack of LmVASH affected the growth rate of amastigotes in cell culture, we tested the multiplication rate of KO cells in macrophages. To this end, we infected differentiated THP-1 macrophages with either parental, null mutant or add-back axenic amastigotes differentiated for 3 d and analyzed the proliferation rate 2 d postinfection (Fig. 3*C*). We found that the growth rate of the KO cells inside macrophages was strongly impaired, as shown by the significant reduction in the parasitic index (PI) as compared to parental or add-back cells. Next, we tested the pathogenicity of LmVASH null mutant in a mammalian host using a mouse model of cutaneous leishmaniasis (Fig. 3*D*). We found that LmVASH null mutant parasites did not form significant lesions as compared to the parental and add-back cell lines (Fig. 3*E*). At the end of the time course, we quantified the number of parasites in the footpads and found a dramatic drop of parasite burden in the KO cell line, with LmVASH add-back partially rescuing the parasite numbers (Fig. 3*F*). Taken together, in the absence of detyrosination, *L. mexicana* parasites showed a reduced proliferation rate in macrophages in vitro and exhibited a drastic drop of pathogenicity and virulence in vivo. Furthermore, these data suggest that detyrosination is absolutely required for efficient infection of the mammalian host.

### Lack of Detyrosination Impacts Morphogenesis and Flagellar Pocket Shape.

**Fig. 4.**
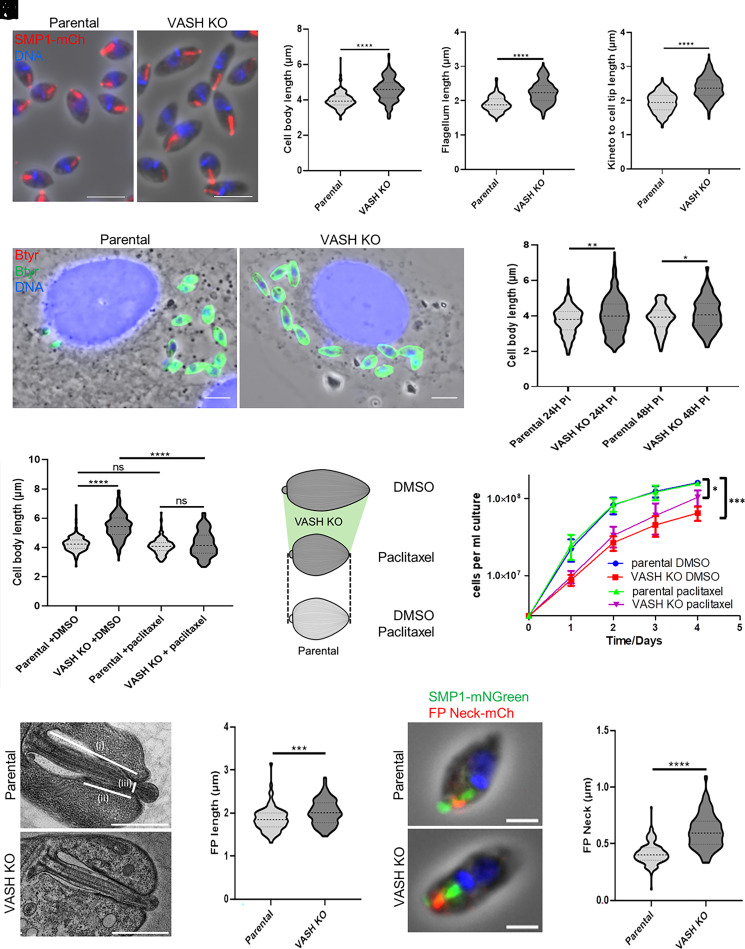
Lack of detyrosination impacts morphogenesis and flagellar pocket shape. (*A*) Fluorescence micrographs of axenic amastigotes cultured over 7 d after differentiation from parental and LmVASH knockout cells expressing SMP1-mCherry. Note the longer flagellum and cell body in LmVASH knockout cells. (Scale bar: 5 µm.) (*B*–*D*) Measurements of cell body, flagellum, and kinetoplast to cell body end distances in parental and LmVASH knockout axenic amastigotes (n = 240,170 and 230 cells, respectively, from three independent experiments for each cell line). (*E*) Immunofluorescence micrographs of THP1 cells infected with parental and LmVASH knockout amastigotes probed with βtyr antibody. (Scale bar: 5 µm.) Note the longer cell body in LmVASH knockout cells. Red and green channels represent extracellular (absent in this picture) and intracellular parasites, respectively. (*F*) Cell body length measurements of intracellular amastigotes from parental and LmVASH knockout cells after 24H and 48H postinfection (PI) inside THP1 macrophages (n = 300 at 24 h from three independent experiments and n = 150 at 48 h from two independent experiments for each cell line). (*G*) Cell body length measurements of axenic amastigotes from parental and LmVASH knockout cells cultured over 6 d after differentiation and treated during 24 h with 200 nM paclitaxel (n = 200). Cells were fixed with PFA at a density of 4 × 10^7^ cells/mL. n indicate cells from three independent experiments for each cell line. Dotted lines indicate the median, upper, and lower quartiles. ****P < 0.0001 (ANOVA-one way). (*H*) Cartoon summarizing the effect of dynamic instability in the cell morphology of amastigotes from LmVASH knockout cells. Paclitaxel-induced microtubule stability restores cell body length in the LmVASH knockout with neutral effects on the parental cell line. Not to scale. (*I*) Growth curve (log scale) of axenic amastigotes from parental and LmVASH knockout cells cultured after 3 d of differentiation and treated during 4 d with 200 nM paclitaxel. Cell density was determined by counting at 24 h intervals (n = 3; error bars represent SD). *P < 0.05, ***P < 0.001 (Unpaired Student *t* test). (*J*) Representative electron micrograph of longitudinal section of the flagellar pocket in parental and LmVASH knockout axenic amastigote cells. (i), (ii), and (iii) represent the flagellar pocket length, flagellar pocket neck lengths and flagellar width at the constriction point, respectively. (Scale bar: 1 µm.) (*K*) Flagellar pocket length measurements of axenic amastigotes from parental and LmVASH knockout cells (n = 75). (*L*) Fluorescence micrographs of axenic amastigotes from parental and LmVASH knockout cells expressing SMP1-mNGreen and the flagellar pocket neck (FP Neck) protein (LmxM.28.1990) fused with mCherry. Note the longer flagellar pocket neck in the LmVASH knockout cell. (Scale bar: 2 µm.) (*M*) Flagellar pocket neck length measurements of axenic amastigotes from parental and LmVASH knockout cells (n = 150). n represents cells from three independent experiments. (*B*–*D*, *F*, *K*, and *M*) Dotted lines indicate the median, upper, and lower quartiles. *P < 0.05, **P < 0.01, ***P < 0.001, ****P < 0.0001 (Mann–Whitney test).

The *Leishmania* cell body undergoes morphological changes during differentiation from promastigote to amastigote and accurate morphogenesis is important for pathogenicity (29, 33, 34). The reduced pathogenicity of LmVASH-deleted parasites in the mouse prompted us to test whether cell morphology was impacted in the absence of detyrosination. We first measured cell body and flagellum lengths in stationary promastigotes akin to parasites used to infect mice (*SI Appendix*, Fig. S5*A*). We observed that both the cell body (*SI Appendix*, Fig. S5*B*) and the flagella (*SI Appendix*, Fig. S5*C*) were significantly shorter in the KO cells as compared to parental promastigotes. Subsequently, we analyzed the amastigote cell morphology that represents the main replicative form in the mouse. Unlike promastigotes, LmVASH-KO amastigotes, cultured over 7 d after differentiation, displayed elongated cell shape as compared to the parental cell line (Fig. 4*A*). Morphometric analysis confirmed that the cell body (Fig. 4*B*) and the flagellum (Fig. 4*C*) lengths were significantly longer in differentiated KO amastigotes. In addition, we observed an increased distance from the kinetoplast to the cell tip which correlates to the length of the internal flagellum within the flagellar pocket (33) (Fig. 4*D*). Importantly, since the observed phenotypes persisted even 2 wk after differentiation, they were not simply the result of delayed cytoskeletal remodeling caused by impaired growth in the amastigote form (*SI Appendix*, Fig. S5*D*). Furthermore, transcript analysis of molecular markers differentially expressed in the amastigote form, such as the up-regulated amastin-like gene (35), the amastigote-specific P1/S1 nuclease (36) and the down-regulated PF16 gene (37), confirmed stage conversion of the LmVASH-KO promastigotes into amastigotes (*SI Appendix*, Fig. S5*E*). Using a multicolor immunofluorescent assay (38), we investigated whether the altered size observed in axenic LmVASH-KO amastigotes persisted within THP-1 infected cells (Fig. 4*E*). After 24 and 48 h postinfection, intracellular knockout amastigotes displayed a significantly longer cell body length compared to the parental cell line (Fig. 4*F*).

Next, we investigated the cause of impaired morphogenesis in the LmVASH null mutant. Recent study suggested that cell morphogenesis in *Leishmania* requires microtubule stability during the differentiation from promastigotes into amastigotes (39). Given that in mammals detyrosination indirectly promotes microtubule stability (40), the lack of this modification might have led to increased microtubule dynamics, thereby affecting cell morphology. We reasoned that in such case, cell treatment with paclitaxel, a microtubule-stabilizing drug, would restore the abnormal cell morphologies observed in LmVASH-KO promastigotes and amastigotes. To test this hypothesis, we used a concentration of paclitaxel that was significantly lower than previously reported IC50 in *L. mexicana* (41) and as such had no effect on the proliferation rate of the parental or LmVASH-KO cells (*SI Appendix*, Fig. S5*F*). Strikingly, a 24-h treatment with paclitaxel restored the cell morphology in both promastigotes (*SI Appendix*, Fig. S5 *B*, *C*, and *G*) and amastigotes (Fig. 4 *G* and *H*) of the LmVASH-KO cell line, while the morphology of the parental cells remained unaffected. Next, we tested whether paclitaxel treatment could restore the growth defect observed in the LmVASH-KO amastigotes after 3 d of differentiation (Fig. 3*B*). A single dose treatment with 200 nM paclitaxel partially restored the growth rate in the LmVASH-KO amastigotes (Fig. 4*I*). Morphometric analysis of these treated amastigotes further revealed that the length of both the cell body (*SI Appendix*, Fig. S5*H*) and the flagellum (*SI Appendix*, Fig. S5*I*) were restored in the LmVASH-KO cell line. However, the distance between the kinetoplast and the cell tip was only partially reduced (*SI Appendix*, Fig. S5*J*), which may account for the incomplete restoration of growth in treated LmVASH-KO amastigotes (Fig. 4*I*).

The increased distance from the kinetoplast to the cell tip in the LmVASH-KO amastigotes prompted us to investigate whether the flagellar pocket was altered in the absence of detyrosination. Transmission electron microscopy (TEM) analysis of longitudinal sections through the flagellar pocket of parental and LmVASH-KO axenic amastigotes (Fig. 4*J*) revealed a significantly longer flagellar pocket in cells lacking detyrosination (Fig. 4*K*). Furthermore, the length of the flagellar pocket neck (Fig. 4*J* and *SI Appendix*, Fig. S5*K*) and the width of the flagellum at the constriction point (Fig. 4*J* and *SI Appendix*, Fig. S5*L*) were significantly increased in the VASH mutant compared to the parental cell line. To further confirm alterations in the flagellar pocket neck of the VASH mutant, we fused the flagellar pocket neck-resident protein LmxM.28.1990 (LeishTag) to an mCherry tag. Immunofluorescence labeling (Fig. 4*L*) and measurements revealed a significantly elongated flagellar pocket neck in LmVASH-KO amastigotes (Fig. 4*M*).

Altogether, these data indicate that decreased microtubule stability, caused by the absence of detyrosination, results in impaired cell morphogenesis affecting cell body, flagellum, and flagellar pocket lengths.

### Tubulin Detyrosination Promotes the Remodeling of the Amastigote Flagellum.

**Fig. 5.**
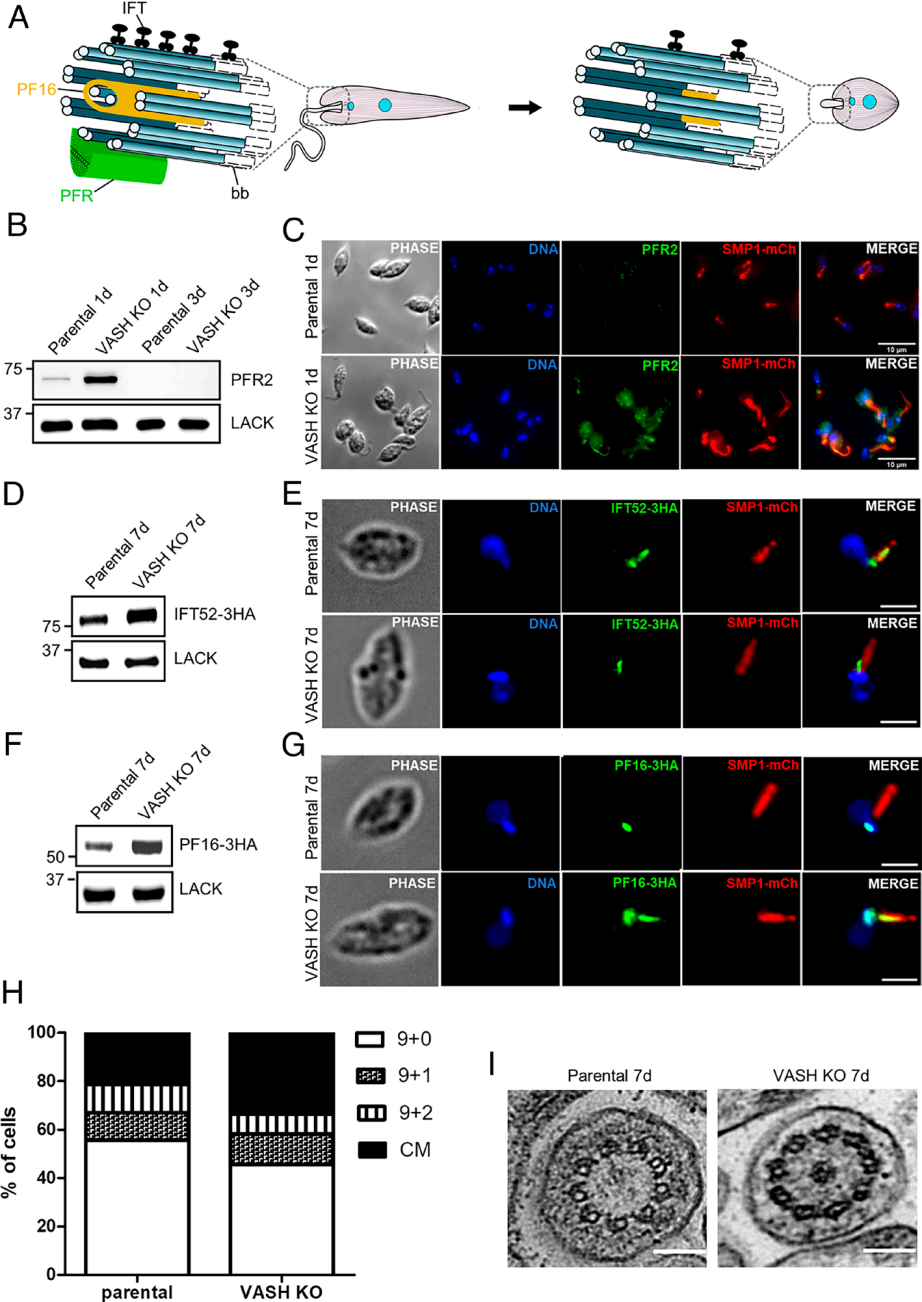
Tubulin detyrosination promotes the remodeling of the amastigote flagellum. (*A*) Schematic overview of the axoneme remodeling from promastigote to amastigote form. Not to scale. (*B*) Western blot of detergent-extracted cytoskeletons showing delayed loss of PFR2 in LmVASH knockout axenic amastigotes compared to parental cells cultured over 1 d (1d) and 3 d (3d) after differentiation. 5 × 10^7^ cytoskeletons were loaded on a 10% SDS-PAGE, transferred and immuno-probed with anti-PFR2 and anti-LACK (loading control) antibodies. (*C*) Immunofluorescence assay of whole cells from parental and LmVASH knockout axenic amastigotes after 24 h postdifferentiation expressing the flagellar membrane marker SMP1-mCh and probed with anti-mCherry and anti-PFR2 antibodies. (Scale bar: 10 μm.) (*D*) Western blot of detergent-extracted cytoskeletons showing increased levels of IFT52-3xHA in LmVASH knockout axenic amastigotes compared to parental cells cultured over 7 d after differentiation. 5 × 10^7^ cytoskeletons were loaded on a 10% SDS-PAGE, transferred, and immuno-probed with anti-HA and anti-LACK (loading control) antibodies. (*E*) Immunofluorescence assay of whole cells from parental and LmVASH knockout axenic amastigotes expressing the flagellar membrane marker SMP1-mCh and IFT52-3xHA. (*F*) Western blot of detergent-extracted cytoskeletons showing increased levels of PF16-3xHA in LmVASH knockout axenic amastigotes compared to parental cells cultured over 7 d after differentiation. 5 × 10^7^ cytoskeletons were loaded on a 10% SDS-PAGE, transferred, and immuno-probed with anti-HA and anti-LACK (loading control) antibodies. (*G*) Immunofluorescence assay of whole cells from parental and LmVASH knockout axenic amastigotes expressing the flagellar membrane marker SMP1-mCh and PF16-3xHA. Cells were probed with anti-mCherry and anti-HA antibodies. (Scale bar: 2 μm.) (*H*) Quantification of 9+0, 9+1, and 9+2 axoneme configurations and central material (CM) in transversal section of transmission electron micrographs from parental (n = 82) and LmVASH knockout (n = 110) axenic amastigotes cultured over 7 d after differentiation. (*I*) Transmission electron micrographs of axenic amastigotes from parental and LmVASH knockout cells cultured over 7 d after differentiation. Transversal sections of the axonemal structure in the parental cell line with a 9+0 configuration and the LmVASH null mutant with nine doublets surrounding a central electron-dense core. (Scale bar: 100 nm.)

In addition to morphological changes, the differentiation of promastigotes into amastigotes also involves a drastic shortening and reorganization of the flagellum. In particular, the axoneme undergoes major restructuring from a motile 9+2 to immotile 9v (variable) organization, which coincides with a rapid loss of the extra-axonemal paraflagellar rod (PFR) (42) (Fig. 5*A*). To test whether flagellar remodeling might be affected by the lack of detyrosination, we analyzed the PFR2 protein, a major component of the PFR. Immunoblot performed on detergent-extracted cytoskeletons showed a delayed loss of PFR2 protein in the LmVASH-deficient cells (Fig. 5*B*). This observation was confirmed by immunofluorescence of amastigotes expressing a SMP1-mCherry as a flagellar marker, revealing a positive PFR2 labeling in LmVASH-KO amastigotes and missing signal in parental cells (Fig. 5*C*). This indicates impaired flagellar remodeling in the absence of detyrosination.

Apart from the removal of PFR, the reconstruction of the flagellum is also associated with a reduced intraflagellar transport (IFT) (42) (Fig. 5*A*), a process involving bidirectional movement of cargo along the axoneme that is essential for the assembly of the flagellum (43). The IFT machinery is composed of two protein complexes, with IFT-B complex transporting cargo in the anterograde direction and the IFT-A complex moving components in the retrograde direction. To investigate the impact of the lack of detyrosination on the IFT machinery, we tagged IFT122 and IFT52, a member of the IFT-A and IFT-B complex respectively, with an HA tag. While immunoblot analysis of detergent-extracted cytoskeletal fractions found no differences in the amount of IFT122 (*SI Appendix*, Fig. S6*A*), the levels of IFT52 were significantly increased in the KO cells as compared to the parental cell line (Fig. 5*D*). However, in contrast to IFT52 expressed in the parental cells, which localized at the basal body and along the flagellum, the protein expressed in LmVASH-deficient cells was found restricted to the basal body region (Fig. 5*E*). Thus, despite increased amounts of IFT52 in the detyrosination deficient cells, it failed to properly distribute along the length of the flagellum.

Next, we analyzed a central-pair-associated protein, PF16, which during the 9+0 reconfiguration of the amastigote axoneme becomes sequestered to the basal body region (42) (Fig. 5*A*). We found not only that the levels of basal-body associated PF16 are increased in the LmVASH-KO cells (Fig. 5 *F* and *G*), but also that it remained present in the flagellum (Fig. 5*G*). This raised the possibility that in the absence of detyrosination, the removal of the central pair might be affected. To test this hypothesis, we performed TEM-based analysis of the central pair distribution in the amastigote axoneme. We found that the majority of the axonemes from the parental cell line showed a 9+0 configuration (56%), while some displayed either 9+1 (11%) or 9+2 (11%) arrangement (Fig. 5 *H* and *I*). In addition, a substantial number of axonemes (21%) was characterized by the presence of an electron-dense core (central material) surrounded by nine doublets. Such distribution was consistent with previous reports (44). In contrast, the KO cells showed a diminished number of axonemes with a 9+0 configuration (45%) (Fig. 5*H*). However, this reduction was not accompanied by a rise in either 9+1 (13%) or 9+2 (8%) arrangements, but instead there was an increased number of axonemes with central material (34%) (Fig. 5 *H* and *I*). Accordingly, the expression levels of highly conserved axonemal structures such as the subunit of nexin-dynein regulatory complex DRC2-HA (*SI Appendix*, Fig. S6*B*) or the member of radial spoke complex RSP4/6-HA (*SI Appendix*, Fig. S6*C*) (42) remained unchanged in LmVASH deficient amastigotes. Taken together, this suggests that the remnants of PF16 in the KO flagella are most likely associated with central material, and not as originally hypothesized with the central pair microtubules. Nonetheless, our results show clear defects in flagellar reorganization caused by the absence of detyrosination.

### Microtubule Depolymerizing Activity of the Flagellar LmKIN13-2 Is Increased in Absence of Microtubule Detyrosination.

**Fig. 6.**
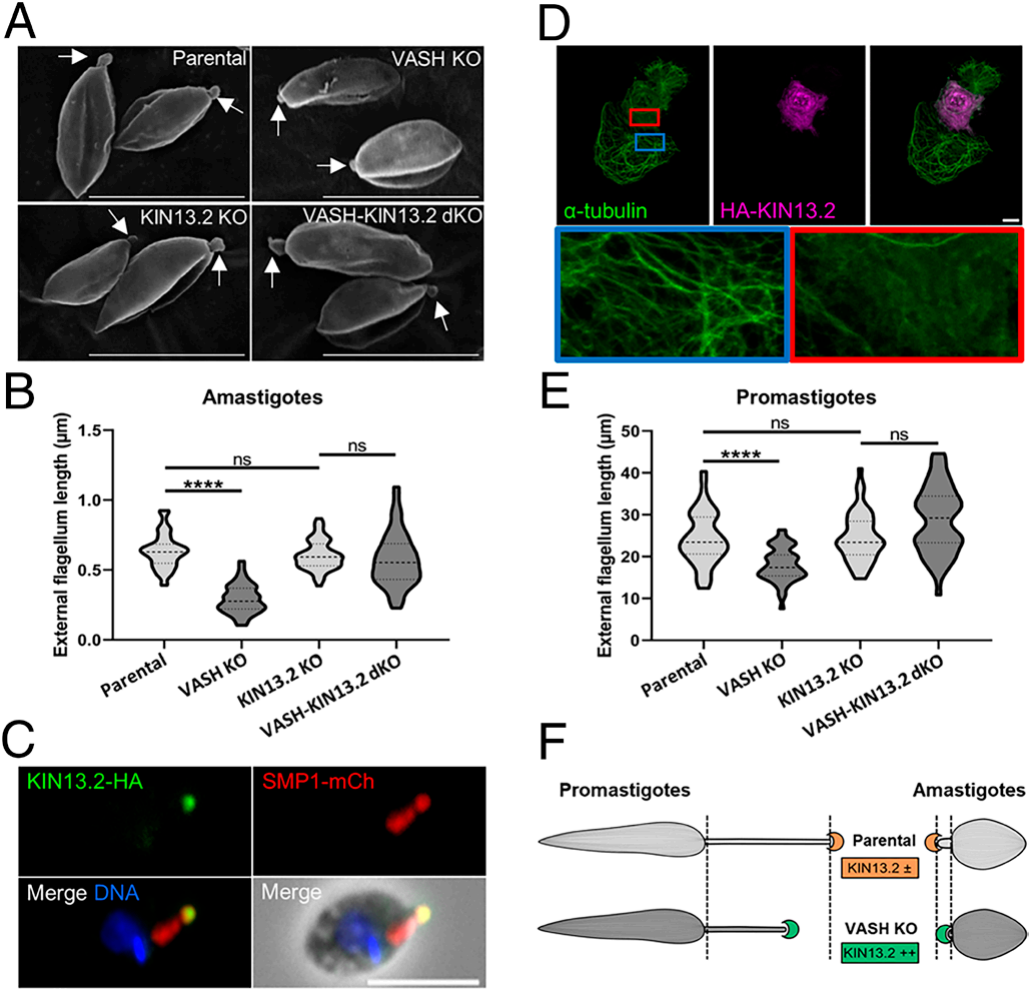
Microtubule depolymerizing activity of the flagellar LmKIN13.2 is increased in the absence of detyrosination. (*A*) Scanning electron microscopy micrographs of axenic amastigotes showing a shorter external flagellum in LmVASH knockout cells compared to parental, LmKIN13.2 knockout and LmVASH-LmKIN13.2 double knockout cell lines cultured over 3 d after differentiation. White arrows show external flagella. (Scale bar: 5 μm.) (*B*) External flagellum length measurements of axenic amastigotes showing that LmKIN13.2 is responsible for shortening of the external flagellum in LmVASH-KO parasites. Measurements were performed on parental, LmVASH knockout, LmKIN13.2 knockout and LmVASH-LmKIN13.2 double knockout cell lines expressing SMP1-mCherry. The external flagellum length corresponds to the distance from the distal end of the SMP1 signal to the cell tip (the flagellum exit point from the cell body). Amastigotes were cultured over 3 d after differentiation and were fixed with PFA at a density of 3 × 107 cells/mL. Fifty cells were counted per sample (n = 3). Dotted lines indicate the median, upper, and lower quartiles. ****P < 0.0001 (ANOVA-one way). (*C*) Immunofluorescence showing localization of LmKIN13.2-3xHA to the flagellum tip in axenic amastigote. Cells coexpressing HA-tagged LmKIN13-2 and SMP1-mCherry were probed with anti-mCherry and anti-HA antibodies. (Scale bar: 5 μm.) (*D*) Microtubule-depolymerizing activity of the LmKIN13.2. Immunofluorescent analysis of tubulin in RPE1 cells expressing LmKIN13-2-HA. Blue and red squares show microtubules and depolymerized tubulin, respectively. (Scale bar: 10 μm.) (*E*) External flagellum length measurements of stationary phase promastigotes showing that LmKIN13.2 is responsible for shortening of the external flagellum in LmVASH-KO parasites. Measurements were performed on parental, LmVASH knockout, LmKIN13.2 knockout and LmVASH-LmKIN13.2 double knockout cell lines expressing SMP1-mCherry. The external flagellum length corresponds to the distance from the distal end of the SMP1 signal to the cell tip (the flagellum exit point from the cell body). Promastigotes were fixed with PFA at a density of 1.7 × 107 cells/mL. Fifty cells were counted per sample (n = 3). Dotted lines indicate the median, upper, and lower quartiles. ****P < 0.0001 (ANOVA-one way). (*C*) Immunofluorescence showing localization of LmKIN13.2-3xHA to the flagellum tip in axenic amastigote. Cells coexpressing HA-tagged LmKIN13-2 and SMP1-mCherry were probed with anti-mCherry and anti-HA antibodies. (Scale bar: 5 μm.) (*D*) Microtubule-depolymerizing activity of the LmKIN13.2. Immunofluorescent analysis of tubulin in RPE1 cells expressing LmKIN13-2-HA. Blue and red squares show microtubules and depolymerized tubulin, respectively. (Scale bar: 10 μm.) (*F*) Schematic representation of the flagellum shortening in the promastigote and amastigote form due to increased activity of the microtubule depolymerizing activity of the flagellar LmKIN13.2 in the absence of detyrosination. Not to scale.

The transition from promastigotes to amastigotes is associated with extensive shortening of the external flagellum, which barely emerges from the anterior cell body in the mammalian form. The impaired morphogenesis (Fig. 4*A*) and flagellar remodeling (Fig. 5) in the LmVASH KO amastigotes prompted us to further investigate whether detyrosination is involved in the shortening of the amastigote external flagellum. Since LmVASH-KO amastigotes displayed longer flagellum after 7 d postdifferentiation (Fig. 4*C*), we asked whether the length of external flagellum was also affected by the lack of detyrosination at earlier stages of differentiation. Strikingly, using light and scanning electron microscopy, we found that after 3 d postdifferentiation, the outer part of the flagellum in LmVASH-KO amastigotes was considerably shorter than that of control cells (Fig. 6*A* and *SI Appendix*, Fig. S7*A*). Morphometric analysis confirmed a significantly shorter external flagellum in the LmVASH-KO amastigotes (Fig. 6*B*). This, however, was associated with an increased distance from the kinetoplast to the cell tip (*SI Appendix*, Fig. S7*B*). These data further confirmed that the loss of LmVASH results in defective morphogenesis in amastigotes and that detyrosination might be involved in flagellar length control.

Among the factors controlling flagellum length in *Leishmania,* the KIN13.2, a member of the microtubule-depolymerizing kinesin-13 family, localizes to the tip of the flagellum and its overexpression leads to flagellar shortening in promastigotes (45). This, together with studies on a mammalian homolog of kinesin-13 that showed its preference for tyrosinated microtubules (8, 46), led us to hypothesize that the shorter flagella observed in the LmVASH-KO cells is associated with increased activity of LmKIN13.2. To test this hypothesis, we first confirmed the localization of the endogenously tagged LmKIN13.2 to the tip of the flagellum in both promastigotes (*SI Appendix*, Fig. S7*C*) and differentiated amastigotes (Fig. 6*C*). Subsequently, to assess the depolymerizing activity of LmKIN13.2, we used a fluorescence microscopy-based assay in mammalian cells (Fig. 6*D* and *SI Appendix*, Fig. S7*D*). Ectopic expression of LmKIN13.2 in RPE1 (Fig. 6*D*) and U2OS (*SI Appendix*, Fig. S7*D*) cells led to the disassembly of organized microtubule structures, as indicated by a diffuse tubulin signal. Quantification showed a significant increase in the number of RPE1 cells with depolymerized microtubules, further confirming LmKIN13.2 depolymerizing activity (*SI Appendix*, Fig. S7*E*). Taken together, these results suggest that LmKIN13.2 regulates flagellar length in response to elevated tubulin tyrosination levels at the flagellar tip, leading to shorter external flagella in LmVASH-KO parasites.

To directly assess the involvement of KIN13.2 in the regulation of flagellar length, we deleted the corresponding gene in the parental and LmVASH-KO cell lines. We first confirmed deletion of the LmKIN13.2 gene in the promastigote cell line by PCR (*SI Appendix*, Fig. S7*F*). In agreement with our initial observations, the mean length of external flagellum in LmVASH-KO mutant cells, whether measured in promastigotes (Fig. 6*E*) or differentiated amastigotes (Fig. 6*B*), was significantly reduced as compared to parental cells. Strikingly, while the external flagellum length was not impacted by the deletion of LmKIN13.2 in parental cells, it was restored in cells lacking both LmVASH and LmKIN13.2 (Fig. 6 *B* and *E*). The amastigote double KO cell line indeed showed external flagella (Fig. 6*A* and *SI Appendix*, Fig. S7*A*) with similar lengths as compared to the parental KIN13.2 KO cells (Fig. 6*B*). Moreover, the increased length of the internal flagellum (distance from kinetoplast to cell tip) in the LmVASH-KO amastigotes remained unchanged in the double KO cell line, indicating that this increase is independent of KIN13.2 activity (*SI Appendix*, Fig. S7*B*). Altogether, our data support the model that KIN13.2 perturbs the length of external flagella in both LmVASH-KO promastigotes and amastigotes due to an increased activity at flagellar tips caused by higher levels of tubulin tyrosination (Fig. 6*F*). These findings highlight an interplay between tubulin detyrosination and microtubule depolymerization in *Leishmania*, establishing the kinesin-13 homolog, KIN13.2, as a functionally relevant reader of the tubulin code.

## Discussion

The tubulin code, allowing a single cell to have distinct populations of microtubules at a given time, is of particular significance in trypanosomatids. These parasites essentially rely on microtubules to accommodate extensive morphological changes throughout their cell and life cycles. While most eukaryotes possess various α- and β-tubulin isotypes with distinct polymerization kinetics and expression patterns, trypanosomatids typically encode a single isotype of each α- and β-tubulin (16). Consequently, tubulin PTMs emerge as the primary regulatory mechanism controlling functional diversification of the parasite’s cytoskeleton. In this study, we highlighted the pivotal role of tubulin detyrosination in regulating *Leishmania* cytoskeletal architecture, morphogenesis, and pathogenesis.

An interesting feature of trypanosomatids is the presence of a C-terminal tyrosine on β-tubulin, suggesting a unique regulatory mechanism within their tubulin code. We have previously demonstrated that the tyrosine residue from both α- and β-tubulin tails in *T. brucei* parasites is removed by a single autonomous VASH enzyme (22). Using a detyrosination assay with heterologous α-tubulin, we found that TbVASH activity is restricted to microtubules. Here, we uncovered distinct enzymatic mechanisms for tubulin detyrosination in *L. mexicana*. Through the analysis of LmVASH detyrosinase activity toward its endogenous α- and β-tubulin substrates, we show that in addition to microtubules it also modifies tubulin dimers, although at a slower rate (Fig. 2*D*). Furthermore, we demonstrate that LmVASH preferentially modifies α-tubulin as compared to β-tubulin (Fig. 2*E*). These findings imply an intricate regulatory process governing tubulin detyrosination in *Leishmania*, which may also extend to *T. brucei* and *T. cruzi* parasites. Despite the absence of SVBP cofactor, LmVASH exhibited robust detyrosinase activity. This parallels the specific activities of its human counterparts, HsVASH2 and HsVASH1 bound to SVBP (22). VASH activity toward both α- and β-tubulin tails in trypanosomatids could be explained by the noncanonical C-terminal tyrosine and third-last glutamate residues in *T. brucei* (−EQY) and *L. mexicana* (−EAY) β-tubulin isotypes (Fig. 1*A*) akin to the canonical residues on α-tubulin crucial for HsVASH1 activity (11). Cryogenic electron microscopy studies have shown that the catalytic site of HsVASH1 is engaged by the α-tubulin tail (47). Likewise, both α- and β-tubulin tails should engage LmVASH catalytic site and form a productive enzyme–substrate docking complex. However, the presence of an alanine positioned between the last tyrosine and second-last glutamate residues (Fig. 1*A*) could hinder β-tubulin recognition by LmVASH, thereby explaining the slower detyrosination kinetics as compared to α-tubulin. Additionally, HsVASH1 establishes multiple contacts with the globular domain of polymerized α-tubulin (47). The absence of specific interacting motifs within the globular domain of β-tubulin may underline the substrate preference of LmVASH toward α-tubulin.

Differential detyrosination of α- and β-tubulin was correlated with data obtained by expansion microscopy which revealed uneven distribution of tyrosinated microtubules in key *Leishmania* structures such as the mitotic spindle and flagellum. Given the activity of LmVASH toward free tubulin dimers (Fig. 2*D*), the spindle may be partially assembled from already modified α- and/or β-tubulin and could undergo a rapid postassembly detyrosination exclusively on α-tubulin, as previously suggested for *T. brucei* (24). The heterogeneous levels of tyrosinated α- and β-tubulin within the old flagellum of dividing promastigotes (Fig. 1*E*) suggest either distinct kinetics of tubulin detyrosination and/or a differential retyrosination for each tubulin subunit. Although the religation of the C-terminal tyrosine by TTL was shown to be restricted to α-tubulin and absent from flagellar microtubules in *T. brucei* (21), the tubulin tyrosination cycle of *Leishmania* parasites may be more complex. In agreement, based on sequence homologies, two TTLs have been identified in the genome of *L. major* (48), while most eukaryotes, including *T. brucei*, possess a single TTL. Thus, the presence of multiple TTLs, potentially associated with distinct retyrosination kinetics and substrate specificity, may further accentuate the complexity of the tubulin tyrosination cycle in *Leishmania*.

Trypanosomatid microtubules are highly glutamylated (21). Polyglutamylation involves the enzymatic addition of glutamate residues to the C-terminal tails of α- and/or β-tubulin by tubulin tyrosine-like ligases (TTLLs) (2). Unexpectedly, the loss of tubulin detyrosination resulted in a strong decrease of the levels of tubulin glutamylation in both *L. mexicana* (Fig. 2*C*) and *T. brucei (**SI Appendix*, Fig. S3*D*). This revealed the cross-talk between the two modifications showing that detyrosination acts as a positive regulator of polyglutamylation. In agreement, in vitro studies involving peptides corresponding to either tyrosinated or detyrosinated C-terminal tails of α-tubulin showed that TTLL6 polyglutamylase has a much higher activity toward the peptide lacking the very C-terminal tyrosine (49). One way in which detyrosination could increase the level of polyglutamylation is simply by unmasking the primary glutamate sequence, which could then be elongated. Alternatively, the polyglutamylating enzymes might preferentially bind to detyrosinated microtubules, thus acting as effectors of this modification. Considering that apart from PolyE, also the signal for GT335 which recognizes the branching point glutamates is reduced, it favors the later hypothesis. Nonetheless, considering the important role of polyglutamylation in the regulation of microtubule cytoskeleton in *T. brucei* (50, 51), it raises the possibility that a reduction in the levels of this modification might have contributed to the observed phenotypes. Furthermore, the crosstalk between these tubulin PTMs demonstrated here in trypanosomatids, is highly likely to also take place in other organisms making it a general mechanism involved in the regulation of microtubule functions.

An unusual feature in *Leishmania,* as compared to other trypanosomatids, is the presence of a distinct single β-tubulin gene on chromosome 21 (26). This β2 isotype exhibits a variable C-terminal sequence as compared to the main β1-tubulin array (Fig. 1*A*) and is upregulated as promastigotes transition into amastigotes (20, 52). The differential expression of β-tubulin isotypes, both encoding a noncanonical C-terminal tyrosine, adds further complexity to the regulatory mechanisms of tubulin detyrosination in *Leishmania*. The substitution of the glutamate residue by a noncharged glutamine in the C-terminal sequence of the amastigote-specific β2 isotype (-QEAY vs. EEAY) could modulate substrate recognition and processing by LmVASH. The resulting changes in the biochemical properties of the β2 isotype tail may have impaired the recruitment of amastigote-specific MAPs to distinct microtubule subcompartments. Indeed, it has been established that variations in the α-tubulin tail primarily influence the recruitment of MAPs rather than changing intrinsic microtubule properties (40). Accordingly, while we observed shortened stationary-phase promastigotes lacking detyrosination (*SI Appendix*, Fig. S5*A*), LmVASH deletion conversely generated elongated amastigotes (Fig. 4*A*). These contrasting phenotypes suggest that the expression of a stage-specific tubulin isotype, potentially subjected to differential modifications and recruitment of variable MAPs, provides tunable properties for the microtubule polymer during *Leishmania* morphogenesis.

As opposed to the highly dynamic tyrosinated microtubules present in mammalian cells, the cytoskeleton of trypanosomatids, is characterized by highly stable microtubules (16). Furthermore, microtubule stability is required for *Leishmania* cell morphogenesis during the differentiation from promastigotes into amastigotes (39). Inducing microtubule stabilization, through pharmacological treatment with paclitaxel, restored the long and short morphologies of LmVASH knockout amastigotes and promastigotes, respectively, without impacting the shape of parental cells (Fig. 4*H* and *SI Appendix*, Fig. S5*G*). The hypersensitivity of mutant cells to paclitaxel indicated that tubulin tyrosination increases microtubule dynamics in *Leishmania*. The fact that cell morphology is distinctively impacted by dynamic instability could be related to the β-tubulin isotypes conferring distinct microtubule dynamics and thus different effects of microtubule-stabilizing drugs. This is consistent with paclitaxel binding to β-tubulin and acting differently on different human tubulin isotypes (53).

In contrast to promastigotes (Fig. 3*A*), altered microtubule dynamics in LmVASH-KO was associated with reduced amastigotes proliferation (Fig. 3*B*), suggesting a greater requirement for detyrosination in the mammalian stage. This may contribute to the drastic drop of pathogenicity in mice (Fig. 3*F*). Particularly, the increased remodeling of the external flagellum tip (Fig. 6*B*) might compromise the proposed sensory function of the amastigote flagellum within macrophages (44). These structural alterations could indeed impair the flagellum’s ability to effectively sense and respond to host environmental signals or deliver parasite effectors necessary for immune evasion and nutrient acquisition. Flagellum restructuring during differentiation from promastigote to amastigote is associated with decreased IFT (54). The altered composition of the 9v axoneme with accumulation of central material and flagellar components such as PF16 and IFT52 (Fig. 5) is consistent with tubulin detyrosination promoting the *Leishmania* flagellum restructuring. Moreover, the tubulin code has been proposed as a regulatory mechanism, restricting IFT movement to specific microtubule doublets in the motile 9+2 flagellum of *T. brucei* (55). Given the presence of a motile 9+2 flagellum in promastigote cells coupled with the altered directionality and flagellum shortening in the LmVASH null mutant, it is plausible that the lack of detyrosination has impacted IFT. These flagellum-related phenotypes may disturb the ability of parasites to infect the mammalian host as previously reported for the *Leishmania* FAZ5 mutant showing decreased directionality and shortened flagellum (33). Moreover, deletion of FAZ5 caused a perturbation of the amastigote flagellar pocket architecture which contains the short protruding amastigote flagellum. Similarly, deletion of LmVASH resulted in alteration of the flagellar pocket architecture (Fig. 4) which might have contributed to the reduced virulence. In particular, the wider flagella at the constriction point (*SI Appendix*, Fig. S5*L*) may have impacted flagellar pocket sealing, making parasites more susceptible to the exposure of detrimental host factors or limit their ability to sequester nutrients (33, 34). In addition, the longer flagellar pocket was correlated with elongated cell morphology of knockout amastigotes. However, in contrast to the partial restoration of the flagellar pocket length (reflected by the distance between kinetoplast to cell tip), elongated cell bodies and flagella were completely restored upon paclitaxel treatment. This suggests that microtubule stability is one of the several intricate factors regulating *Leishmania* morphogenesis.

Dynamic instability, in the absence of detyrosination, was further supported by the positive regulation of the LmKIN13.2 microtubule depolymerase. The resulting convergent shortening of the external flagellum in both LmVASH-KO amastigotes and promastigotes (Fig. 6) suggests that LmKIN13.2 is a specific effector of tubulin tyrosination, akin to its human MCAK counterpart (8, 46). Surprisingly, deletion of LmKIN13.2 in parental cells did not significantly increase external flagellum lengths (Fig. 6 *B* and *E*). These results indicate that its depolymerase activity is tightly regulated, through detyrosination of the microtubule substrate but also probably through specific PTMs on the enzyme itself, as demonstrated for KIF2A phosphorylation (56), a human ortholog of LmKIN13.2. Our data correlate with recent rescue experiments in RPE-1 cells in which depleting KIF2A restored the reduced length of cilia caused by the depletion of Tau-tubulin kinase 2, a negative regulator of KIF2A activity (56), while having no impact on the growth of parental cilia (57). Similarly, detyrosination restrains the microtubule depolymerizing activity of LmKIN13.2 and potentially influences other members of the Kinesin-13 family in *Leishmania* and related trypanosomatid parasites.

Altogether, this work paves the way for future studies to identify MAPs readers associated to different levels of tyrosination. Some of these effectors may account for the loss of virulence in LmVASH-deleted parasites and could reveal common regulatory mechanisms underlying the impact of the absence of detyrosination in trypanosomatids. To conclude, we emphasize a sophisticated machinery employed by *Leishmania* to modulate cytoskeletal architecture. The finding of distinct enzymatic mechanisms for tubulin detyrosination sheds light on the intricate regulatory processes governing microtubule dynamics in this parasite. These insights open potential avenues for therapeutic intervention targeting tubulin detyrosination as a strategy to impede *Leishmania* pathogenicity.

## Materials and Methods

### Cell Culture and Generation of Transgenic Lines.

All cell lines, culture, and transfection conditions are detailed in *SI Appendix*.

### DNA Constructs.

Cloning strategies are detailed in *SI Appendix*. Primers used are listed in *SI Appendix*, Table S1.

### Recombinant LmVASH Expression and Purification.

Detailed purification protocol of recombinant LmVASH can be found in *SI Appendix*.

### *Spodoptera frugiperda*-Derived Sf9 Tubulin Purification and Polymerization.

Detailed purification protocol of SF9 tubulin is provided in *SI Appendix*. To get microtubules, tubulin was polymerized in the presence of 1 mM guanosine triphosphate (GTP) and 20 μM paclitaxel for 40 min. Microtubules were centrifugated for 30 min at 220,000 g and resuspended in BRB80, 1 mM dithiothreitol (DTT), 1 mM GTP, 20 μM paclitaxel.

### *Leishmania* Tubulin Purification.

Purification of tubulin from the parental and LmVASH KO promastigotes was conducted following previously published protocols with some modifications (28, 58) (*SI Appendix*).

### In Vitro Characterization of LmVASH Detyrosination Activities on *Spodoptera frugiperda*-Derived Sf9 or LmVASH-KO Tubulin and Microtubules.

For each time point, 4 μg of tubulin or microtubules purified from SF9 or LmVASH-KO cells were incubated at 30 °C with 2 μg of recombinant LmVASH in a total of 160 μL assay buffer (50 mM Tris pH7.4, 10% Glycerol, 2 mM DTT). For tubulin, nocodazole was added to the reaction at 100 ng/mL to avoid polymerization. For microtubules, paclitaxel was added at 20 μM to avoid depolymerization during the reaction. At the end of the reactions, 40 μL of Laemmli buffer 5× was added and the samples were denaturated at 95 °C for 5 min. Reactions were analyzed by sodium dodecyl-sulfate polyacrylamide gel electrophoresis (SDS-PAGE) followed by western-blot as previously described (22). Primary antibodies are detailed in *SI Appendix*. Immunoblots were quantified using Image J.

### Standard Immunofluorescence Staining.

Immunofluorescence labeling was performed as previously described (29) and is detailed in *SI Appendix*.

### Ultrastructure Expansion Microscopy.

The protocol was adapted from ref. 59 (*SI Appendix*). Expanded parasites were imaged with the Zeiss LSM880 confocal microscope equipped with the Airyscan detector, with a 63× oil objective NA = 1.4, using Zen Black software (Zeiss, Intelligent Imaging Innovations).

### Western Blot Analysis.

For western blotting analysis, *Leishmania* extracted cytoskeletons were separated by SDS-PAGE and transferred onto PVDF membranes (Hybond-P, Amersham). Membranes were incubated overnight at 4 °C with specific primary antibodies (*SI Appendix*) diluted in blocking solution.

### Macrophage Infections and Multicolor Immunofluorescence Assay.

THP-1 cells in the log phase of growth were differentiated in Labtek chamber slides or 12 mm coverslip at a concentration of 5 × 10^4^ cells/well and 1 × 10^5^ cells/coverslip, respectively, and incubated for 3 d in medium containing 20 ng of phorbol myristate acetate/mL. THP-1 cells treated with PMA were infected with axenic amastigotes at a parasite/macrophage ratio of 5:1 and 15:1 for PI and multicolor immunofluorescence assay, respectively. Cells were incubated for 4 h at 37 °C with 5% CO_2_. Noninternalized parasites were removed. After 2 d of incubation, cells were fixed with methanol and stained with Giemsa. The PI was defined as the percentage of infected macrophages × number of intracellular parasites/macrophage. Multicolor immunofluorescence assay for cell body measurements of intracellular amastigotes was performed as previously described (38) after 24H and 48H postinfection. Cells were probed with rabbit anti-Leishmania-β- tyr-tubulin, 1:10,000 dilution.

### Infection of Mice.

Experiments involving mice were conducted according to the Animals (Scientific Procedures) Act of 1986, United Kingdom, and had approval from the University of York Animal Welfare and Ethical Review Body committee. Strain virulence was assessed by footpad swelling and parasite burden (60). Detailed protocol is provided in *SI Appendix*.

### Scanning and Transmission Electron Microscopy.

Cells were fixed for 2 h at room temperature with 2.5% glutaraldehyde in 0.1 M cacodylate buffer (pH 7.4), and then stored at 4 °C until further processing. Detailed protocol for scanning electron microscopy and transmission electron microscopy are provided in *SI Appendix*.

### Quantification and Statistical Analysis.

Cell body and flagellum length measurements were performed using Zen Blue 3.6 (Zeiss) software. Statistical analyses were performed using Student’s *t* test and one-way ANOVA. Data are represented as means ± SE of the mean (SEM) or SD; n indicates the number of individual experiments otherwise mentioned. *P* values are indicated within figure legends and in figures as follows: * < 0.05, ** < 0.01, *** < 0.001. Numbers of counted cells are indicated within figure legends when required. Graphical illustrations and statistical analyses were performed using Prism software (GraphPad Software, Version 8.3).

## Supplementary Material

Appendix 01 (PDF)

Movie S1. Confocal z-stack images from expansion microscopy of *Leishmania* promastigotes labelled with αΔ1 and YL1/2 antibodies.

Movie S2. Confocal z-stack images from expansion microscopy of *Leishmania* promastigotes labelled with αΔ1 and YL1/2 antibodies.

Movie S3. Confocal z-stack images from expansion microscopy of *Leishmania* promastigotes labelled with β1Δ1 and YL1/2 antibodies.

Movie S4. Confocal z-stack images from expansion microscopy of *Leishmania* promastigotes labelled with βtyr and YL1/2 antibodies.

Movie S5. Confocal z-stack images from expansion microscopy of *Leishmania* promastigotes labelled with βtyr and YL1/2 antibodies.

## Data Availability

All study data are included in the article and/or supporting information.
