# Supplementary material for: Tubulin detyrosination shapes Leishmania cytoskeletal architecture and virulence
Source: Proc Natl Acad Sci U S A. 2025 Jan 14;122(3):e2415296122. doi: 10.1073/pnas.2415296122 (PMC11761321; doi:10.1073/pnas.2415296122)
Supplement: Supplementary file 1 — Appendix 01 (PDF) [file pnas.2415296122.sapp.pdf]

PNAS

Supporting Information for

Tubulin detyrosination shapes *Leishmania* cytoskeletal architecture and virulence

Rosa Milagros Corrales[a], Jeremy Vincent[a,1], Lucien Crobu[a,1], Rachel Neish[b], Binita Nepal[c,d], Julien Espeut[e], Grégoire Pasquier[a], Ghislain Gillard[e], Chantal Cazevieille[f], Jeremy C Mottram[b], Dawn M Wetzel[c,d], Yvon Sterkers[a], Krzysztof Rogowski[e], Maude F Lévêque[a,2]
*These authors contributed equally

[a]MiVEGEC, University of Montpellier, CNRS, IRD, 34095 Montpellier, France
[b]York Biomedical Research Institute, Department of Biology, University of York, York, United Kingdom
[c]Department of Pediatrics, University of Texas Southwestern Medical Center, Dallas, TX 75390, United States
[d]Department of Biochemistry, University of Texas Southwestern Medical Center, Dallas, TX 75390, United States
[e]Tubulin Code team, IGH, CNRS, Université Montpellier, 34090 Montpellier, France
[f]Université Montpellier, INSERM U1298, INM, 34091 Montpellier, France

[1]J.V. and L.C. contributed equally to this work.
[2]To whom correspondence may be addressed. Email: maude.leveque@umontpellier.fr.

This PDF file includes:

Supporting text
Figures S1 to S7
Table S1
Legends for Movies S1 to S5
SI References

Other supporting materials for this manuscript include the following:

Movies S1 to S5

Supporting Information Text

Materials and Methods

Cell culture and generation of transgenic lines

Cell culture

Human leukemia monocyte cells (THP-1) were cultured in RPMI 1640 medium (Thermo Fisher) supplemented with 10% FCS, 2 mM glutamine, 100 IU penicillin ml-1 and 100 mg streptomycin ml-1. Cells were cultured at 37°C and 5% CO2. HEK and RPE1 cells were cultured in Dulbecco's modified eagle medium/F-12 GlutaMAXTM-I (Gibco) supplemented with 10% heat-inactivated fetal bovine serum (Gibco) and antibiotics (penicillin/streptomycin) (Gibco). U2OS cells were cultured in Dulbecco's modified eagle medium + GlutaMAXTM-I (Gibco) supplemented with 10% heat-inactivated fetal bovine serum (Gibco) and 1% penicillin/streptomycin (Gibco). Sf9 cells were grown in EX-CELL_ 420 Serum-free medium (14420C, Sigma-Aldrich). Promastigote forms of *L. mexicana* Cas9 T7 strain (1) (derived from *L. mexicana* WHO378 strain MNYC/BZ/62/M379) were grown at 27°C in HOMEM medium supplemented with 0.005% hemin and 10% FCS. For selection and maintenance of genetically modified *L. mexicana Cas9 T7* lines, the relevant selection drugs were added to supplemented HOMEN medium as previously described (1). Axenic amastigotes were generated by subculturing into Schneider's Drosophila medium (Thermo Fisher) supplemented with 20% FCS and 25mM MES-HCl (pH 5.5) at 34°C with 5% CO2.

Transfection of human cells

HEK and RPE1 cells were transfected using jetPEI (Polyplus) according to manufacturer's protocol. HEK cells were transfected with active or dead (C92A mutation) LmVASH and RPE1 cells were transfected with active or dead (C92A mutation) HA-VASH as well as with HA-Kinesin13-2 constructs. Cells were collected 24 hours after transfection for immunoblot and immunofluorescence analysis (2).

Generation of *Leishmania* transgenic lines

Deletion of LmVASH (LmxM.27.0460), KIN13-2 (LmxM.13.0130) and tagging were performed using CRISPR-Cas9 as previously described (27). The online primer design tool www.LeishGEdit.net was used to design primers for amplification of the 5' and 3' sgRNA templates and for amplification of donor DNA from pT, pPLOT and pLPOT plasmids (1, 3).

DNA constructs

LmVASH full length sequence was amplified by PCR and cloned in frame with an N-Term HA or GFP tag using *Mfe*I and *Hpa*I cloning sites. Site-directed mutagenesis was directly performed on pTH6-HA_LmVASH_WT using the In-Fusion HD Cloning Kit (Clontech) with self-complementary primers to obtain an enzymatically LmVASH dead version (PTH6-HA_LmVASH_C92A). LmVASH and LmVASH_C92A were cloned into pCold and pRK5-HA vectors using BamHI/EcoRI cloning sites for bacterial production of recombinant proteins and expression in HEK293 cells, respectively. For expression in RPE1 cells, LmKIN13.2 was cloned in pRK5-HA using BamHI/EcoRI cloning sites. All DNA constructs were verified by DNA sequencing prior to transfection.

Recombinant LmVASH expression and purification

LmVASH coding region was cloned into pCold vector with a polyhistidine tag. BL21 bacteria were transformed, grown at 37°C until OD600 reaches 0.6, cold shocked in ice for 30 minutes and induced with 0.5mM IPTG overnight at 18°C. Bacteria were pelleted and lysed in 50mM Tris-HCl pH 7.4, 500mM NaCl, 2mM TCEP, 20mM Imidazole, 0.1% Tween for 30 minutes at 4°C. The lysate was sonicated 3 times, 3 minutes (10 seconds-10 seconds burst) and centrifugated for 40 minutes at 22,000g. The supernatant was loaded onto a 1ml Ni-NTA agarose column, washed with 50mM Tris-HCl pH 7.4, 500mM NaCl, 2mM TCEP, 20mM Imidazole, 0.1% Tween and eluted with 250mM

Imidazole. The fractions containing LmVASH were pooled, concentrated with Amicon Ultra-4ml 10K device, dialysed in 50mM Tris-HCl pH 7.4, 150mM NaCl, 2mM TCEP, snap frozen and stored at −80°C.

Spodoptera frugiperda-derived Sf9 tubulin purification and polymerisation

SF9 cells were grown and lysed in PMI-DAGN60 buffer (0.1M PIPES pH6.8, 60mM NaCl, 10mM MgSO4, 2mM EGTA, 5mM DTT, 2mM ATP, 1mM GTP, 0.2% NP40) for 40 minutes at 4°C. The lysate was sonicated for 2 minutes (10 seconds-10 seconds burst) and centrifugated for 40 minutes at 22,000g. The supernatant was loaded on a 5ml DEAE-Sepharose column, washed with PMI-DAGN60 buffer and eluted with PMI-DAGN500 buffer (0.1M PIPES pH6.8, 500mM NaCl, 10mM MgSO4, 2mM EGTA, 5mM DTT, 2mM ATP, 1mM GTP). The fractions containing purified SF9 tubulin were pooled, concentrated with Amicon Ultra-4ml 10K device and dialysed in BRB80 buffer (80mM K-PIPES pH 6.8, 1mM MgCl2, 1mM EGTA) + 1mM DTT, 10% glycerol.

Leishmania tubulin purification

Schneider′s Insect Medium (Sigma Aldrich) supplemented with 20% heat inactivated FBS (Gemini Bio), was used to grow promastigotes from both parental and LmVASH KO for 72 hrs at 26°C as a static culture. Cell pellets made from centrifuging these cultures were harvested and resuspended in buffer A (100 mM PIPES (pH 6.9), 2 mM MgCl2, 1 mM EGTA and 20 µM GTP). Two complete mini EDTA-free protease inhibitor cocktail tablets (Roche), 2 µl of 25 U/µl benzoase nuclease (Millipore) were added. The cell suspension was lysed using an Emulsiflex C5 homogenizer (Avestin). Lysate was clarified by spinning at 40,000 g at 4°C for 30 mins and the supernatant was passed through a 0.45 µm filter. The clarified lysate was loaded in an anion exchange HiTrap Q HP column (5 ml, Cytvia), previously equalized with 5 column volume (CV) of buffer A. The column was then connected to the AKTA fast performance liquid chromatography system (GE) and washed with 5 CV of buffer A. Tubulin was eluted with a gradient of 30-45% of Buffer B (100 mM PIPES (pH 6.9), 2 mM MgCl2, 1 mM EGTA, 1M KCl, 20 µM GTP). Fractions were run on an SDS PAGE gel and stained with Coomassie blue dye. Tubulin-rich fractions were pooled together and concentrated using Amicon ultracentrifugal filters, 30 kDa MWCO. Concentrated protein was then loaded into a previously equilibrated gel filtration column, HiLoad 16/600, superdex, 200 pg (Cytvia). Protein was eluted with 1 CV of buffer A. Fractions were run on an SDS PAGE gel and stained with Coomassie blue dye. The purified tubulin fractions were pooled together, concentrated to ~3 mg/ml, snap frozen in liquid nitrogen, and stored at -80°C.

In vitro characterization of LmVASH detyrosination activities on *Spodoptera frugiperda*-derived Sf9 or LmVASH-KO tubulin and microtubules

Primary antibodies used: anti-Drosophila-αΔ1-tubulin (1:1000), rat anti-tyrosinated α-tubulin YL1/2 (1:1000), rabbit anti-*Leishmania* β_1-Δ1-tubulin (1:5000), rabbit anti-tyrosinated *Leishmania* β-tubulin (1:15000), mouse anti-α-tubulin 12G10 (1:1000), mouse anti-His (1:1000; ProteinTech) antibodies. Protein bands were visualized with HRP conjugated secondary antibodies (Cell Signaling Technology and Merck) and revealed with SuperSignal West Pico PLUS (Thermo Fisher Scientific) using a Chemidoc Touch imaging system (Bio-Rad).

Standard immunofluorescence staining

Immunofluorescence labelling on *Leishmania* cells were performed as previously described (4) with slight modifications. Whole cells were fixed overnight in 2% paraformaldehyde at 4°C, washed in PBS and then spread on poly-lysine–coated slides. For cytoskeleton preparations, cells were first extracted with 0.25% NP40 in PIPES buffer (100 mM PIPES [pH 6.9], 1 mM MgCl2) for 5 min, and then washed twice in PIPES buffer. Cytoskeletons were fixed in 4% paraformaldehyde for 5 min and neutralised for 10 min in 100mM Glycine, then permeabilized in 0.2% Triton X100 for 10 min. After three washes in PBS, whole cells and cytoskeletons were blocked in 2% bovine serum albumin (BSA) for 30 min. Cells were probed with primary antibodies overnight at 4°C diluted in

blocking solution (rabbit anti-αΔ1 tubulin, 1:2000 dilution; rabbit anti-*Leishmania*-$β_1$Δ1 tubulin, 1:5000 dilution, rabbit anti-β-tyr tubulin, 1:10000 dilution, rat YL1/2 (Chemicon), 1:1000 dilution, rat anti-HA (Roche), 1:200 dilution, mouse anti-PFR2 (2E10B7), 1:1000 dilution, anti-mCherry (Gene Tex), 1:1000 dilution and mouse anti-mNeonGreen (Chromotek) 1:1000 dilution. Slides were washed in 2% BSA before incubation with anti-IgG specific secondary antibodies conjugated to Alexa Fluor 488 (1:2000) or Alexa Fluor 546 (1:2000) (Thermo-Fisher) for 1 h. Slides were washed in PBS, and DNA was stained with Hoechst and mounted with Slowfade gold medium (Invitrogen). Images were acquired using Zen Blue 3.6 (Zeiss) software, on a Zeiss imager Z1 microscope equipped with a Plan-Apochromat 100x/1.40 oil objective and a Hamamatsu digital camera C11440. RPE1 cells were fixed in methanol for 5 min at –20°C. Immunostainings were performed in PBS supplemented with 3% BSA and 0.1% Triton. Primary antibodies used: rabbit anti-αΔ1-tubulin 1:1000, mouse DM1A (anti-α-Tubulin, Sigma, T9026) 1:1000 and rat anti-HA (Roche) 1:500. Secondary antibodies coupled to Alexa-488, Alexa-555 and Alexa-647 from Invitrogen Molecular Probes were used at 1:1000. Optical sections of the cells were acquired with a 63X Plan Apochromat 1.4 NA oil DIC objective on a Leica SP8 confocal equipped with hybrid detectors with a GaAsP photocathode.

Ultrastructure Expansion Microscopy

Leishmania cells from parental and LmVASH KO were loaded on 12-mm coverslips in 24-well plate and cells left to adhere 10 minutes. Cells were then covered with 1 mL of fresh activation solution (4% formaldehyde; 4% Acrylamide in PBS) and incubated overnight at room temperature (RT). For the gelation step, coverslips were gently deposited on top of a 25 µL drop of MS solution (19% Sodium acrylate (AK Scientific); 10% Acrylamide (Euromedex); 0.1% Bis-acrylamide (Euromedex), 0.5% TEMED (Euromedex), 0.5% Ammonium persulfate (Euromedex) in PBS) for 5 min on ice then transferred at 37˚C and incubated for 1 hour in a moist chamber without agitation. The coverslips were then transferred in 6-well plate in 1.5 ml of denaturation solution (200 mM Sodium Dodecyl Sulfate; 200 mM Sodium chloride; 50 mM Tris pH 9.0) with agitation at RT for 15 min to detach the gel from the coverslip, then moved into a 1.5 ml Eppendorf centrifuge tube filled with denaturation solution and incubated at 95˚C for 90 min. Gels were expanded in 50 ml of deionized water (twice 30 min then overnight) then incubated in 50 ml of PBS for 15 minutes (three times). Next, portion of the gels were incubated in blocking solution (PBS, 2% BSA, 0.1% Tween-20) for 60 min at 37˚C. The primary antibodies (rabbit anti- αΔ1-tubulin, 1:500 dilution; rabbit anti-*Leishmania*-$β_1$Δ1-tubulin, 1:1000 dilution, rabbit anti-*Leishmania*-β- tyr-tubulin, 1:750 dilution, rat YL1/2, 1:200 dilution), diluted in blocking solution were incubated overnight in the dark at 37˚C with slow agitation. After three washes in blocking solution, gels were incubated with anti-IgG specific secondary antibodies conjugated to Alexa Fluor 488 (1:500 dilution) or Alexa Fluor 594 (1:500) (Thermo Scientific) diluted in blocking solution with 57 µM Hoechst 33342 (Thermo Scientific) for 3 hours in the dark at 37˚C with slow agitation. After three washes in blocking solution, gels were expanded in 50 ml volume of deionized water (twice 30 minutes then overnight). An expansion factor of 4.3 was determined using the ratio between the size of the coverslip (12 mm) and the size of the gels after the first expansion. Z-stacks were processed to obtain maximum intensity projections with the program Zen Black software.

Western blot analysis

Membranes were incubated overnight at 4°C with specific primary antibodies diluted in blocking solution (mouse anti- α-tubulin 12G10, dilution 1:3000, mouse anti- β-tubulin E7, dilution 1:3000, rabbit anti- αΔ1-tubulin, 1:10000 dilution; rabbit anti- *Leishmania*-$β_1$Δ1-tubulin, 1:5000 dilution, rabbit anti- *Leishmania*-βtyr-tubulin, 1:15000 dilution, rat YL1/2 (Chemicon), 1:2000 dilution, rat anti-HA (Roche), 1:1000 dilution, mouse anti-PFR2 (2E10B7), 1:1000 dilution, rabbit anti-LACK(61), 1:5000 dilution, rabbit anti-Polyglutamate chain polyE (Adipogen), 1:20000 dilution, mouse anti-polyglutamylation GT335 (Adipogen), 1:30000 dilution, anti-acetyl-alpha tubulin, clone 6-11B-1 (Millipore), dilution 1:5000. After washing, membranes were incubated with HRP conjugated secondary antibodies (Biorad) for 1 h at room temperature and revealed with Clarity Western ECL substrate according to manufacturer's instructions.

Motility assay

For the motility assay (4), cells taken from cell cultures at a density of $5x10^6$ cells/ml were used. 5 µL of cell culture were placed on a glass slide covered with a #number 1.5H coverslip and imaged 30 sec at five frames per second using TL bright field illumination with a 10X/0.25 Ph 1 objective. Mean speed and directionality (Velocity/Speed) were measured using the open-source Fiji plugin TrackMate (5).

Fluorescence in situ hybridization (FISH)

Slides and the hybridization protocol were performed as previously described (6). Briefly, amastigotes from parental and LmVASH-KO were fixed in 4% paraformaldehyde and dehydrated in serial ethanol baths (50%–100%). The probe targeting the spliced leader gene, specific for chromosome 2 (6) was labeled with tetramethyl-rhodamine-5-dUTP (Roche Applied Sciences) by using the Nick Translation Mix (Roche Applied Sciences) according to the manufacturer's instructions. Slides were then mounted in Vectashield (Vector Laboratories) with DAPI and microscopically examined. To analyze copy number of chromosomes, three independent experiments were performed and 400 cells were counted in each replicate.

Infection of mice

For experimental infections, the parasites had previously been passaged through mice, isolated, and transformed into promastigotes before re-infection. A high inoculum of 2×10^6 cells from a three-day stationary promastigote culture of each cell line (LmVASH-KO, parental and LmVASH add back) was used to infect the footpad of five individual BALB/c mice. The infections were followed weekly by footpad measurements. Six weeks after challenge, the mice were culled, infected footpads were dissected. The tissues were mechanically dissociated and filtered through a 70 µm cell strainer. Homogenates were resuspended in HOMEN supplemented with 20 % fetal bovine serum, and serial dilutions were performed. These plates were incubated at 25 °C for three weeks and the number of parasites was calculated by multiplying by the dilution factors.

Scanning and transmission electron microscopy

For scanning electron microscopy cells were washed with cacodylate buffer and dehydrated in a graded series of ethanol (30–100%), dried in Hexamethyldisilazane, coated with gold palladium, and observed using a Hitachi S4000. microscope (INM, Montpellier). For transmission electron microscopy, following cell washing after glutaraldehyde fixation, cells were incubated with 0.5% osmic acid, 0.8% potassium Hexacyanoferrate trihydrate for 2H at room temperature in the dark. Then, samples were washed in PHEM buffer, dehydrated using ethanol gradient solutions (30%-100%), and embedded in EmBed 812 using an Automated Microwave Tissue Processor for Electronic Microscopy, Leica EM AMW epoxy resin. Ultrathin (70-nm) cross sections were cut and stained with 1.5% uranyl acetate in 70% Ethanol and lead citrate and observed using a Tecnai F20 transmission electron microscope at 120KV (INM, Montpellier).

Quantitative PCR

Total RNAs were extracted using the RNeasy Mini kit (Qiagen) following the manufacturer's instructions. RNAs were treated with DNase-TURBO (Ambion) for 30 minutes at 37°C and cDNAs were synthesized using the Super Script III cDNA Synthesis Kit (Invitrogen) following the manufacturer's protocol. Quantitative PCR assays were conducted using the LightCycler-480 SYBR Green I master kit (Roche). Reaction mixtures (15µl) were composed according to the manufacturer's protocol. Reactions were carried out in a ROCHE Real-Time LC480 System using the following cycling conditions: 95°C for 5 min, followed by 44 cycles at 95°C for 10s, 58°C for 10s and 72°C for 10s. The relative amount of target cDNA was obtained by normalization using the housekeeping gene GAPDH as internal control.

Fig. S1.

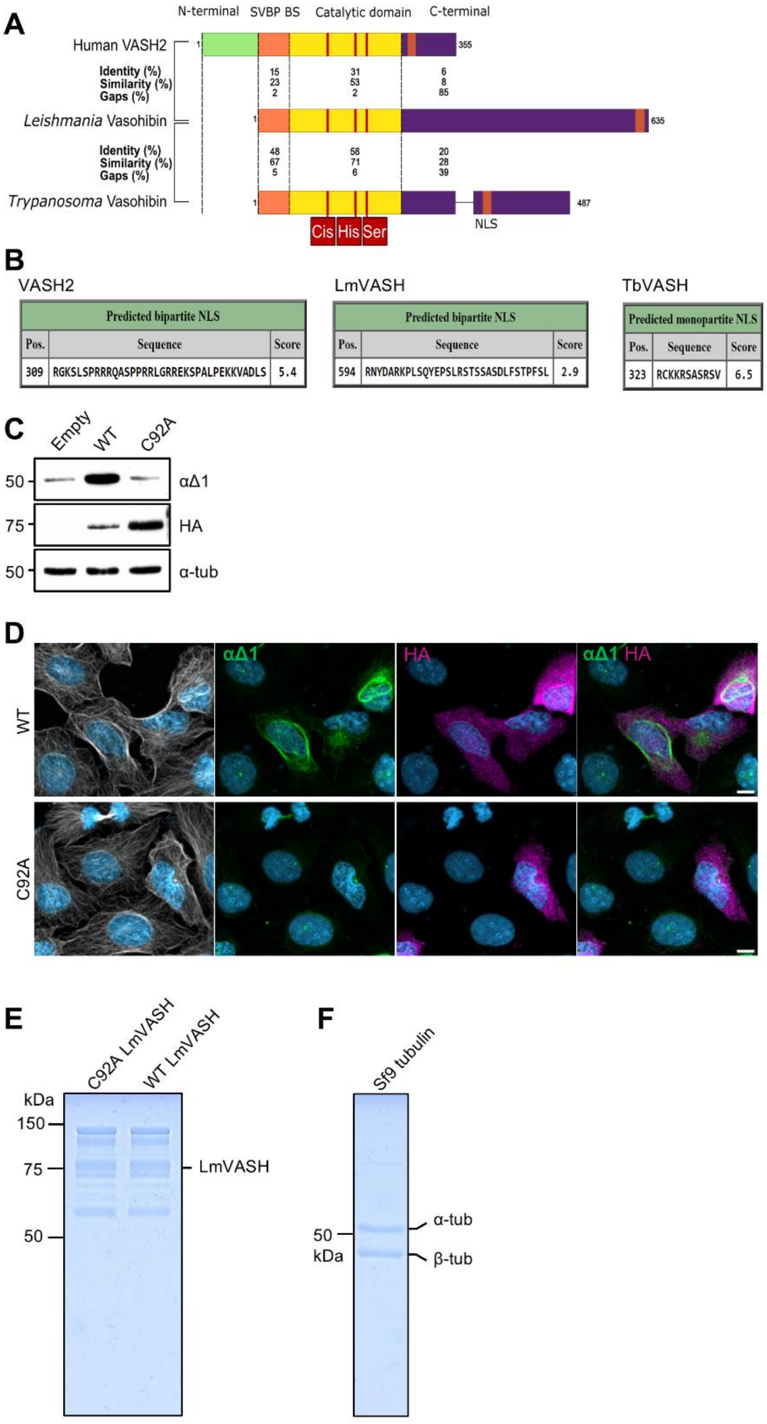
A
N-terminal
SVBP BS
Catalytic domain
C-terminal
Human VASH2
1
355
Identity (%)
Similarity (%)
Gaps (%)
15
23
2
31
53
2
6
8
85
Leishmania Vasohibin
1
635
Identity (%)
Similarity (%)
Gaps (%)
48
67
5
58
71
6
20
28
39
Trypanosoma Vasohibin
1
487
NLS
Cis
His
Ser
B
VASH2
Predicted bipartite NLS
Pos.
Sequence
Score
309
RGKSLSPRRRQASPPRRLGRREKSPALPEKKVADLS
5.4
LmVASH
Predicted bipartite NLS
Pos.
Sequence
Score
594
RNYDARKPLSQYEPSLRSTSSASDLFSTPFSL
2.9
TbVASH
Predicted monopartite NLS
Pos.
Sequence
Score
323
RCKKRSASRSV
6.5
C
Empty
WT
C92A
50
αΔ1
75
HA
50
α-tub
D
WT
C92A
αΔ1
HA
αΔ1 HA
E
C92A LmVASH
WT LmVASH
kDa
150
75
50
LmVASH
F
Sf9 tubulin
50
kDa
α-tub
β-tub

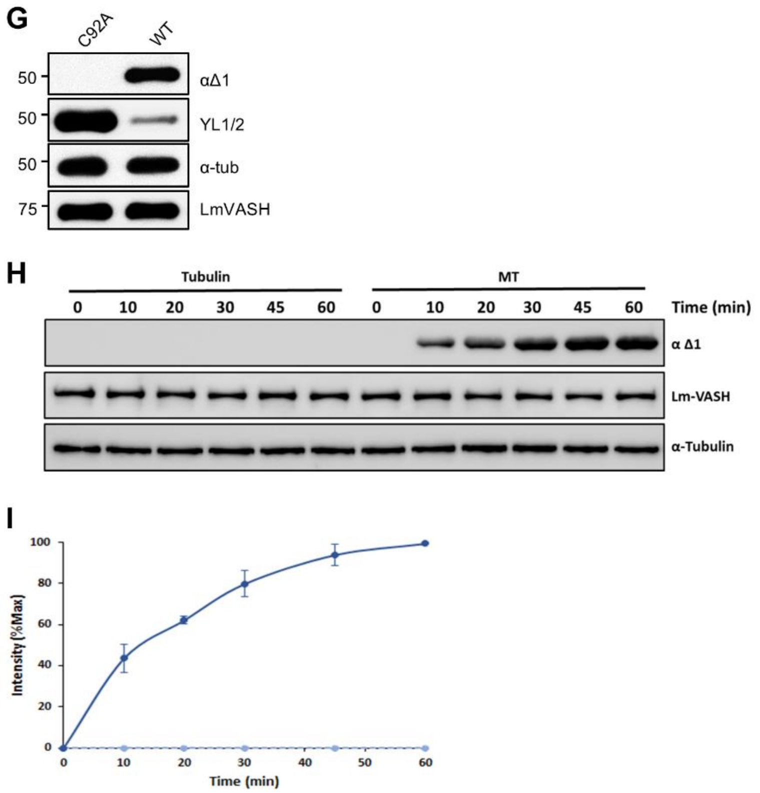

The *Leishmania* VASH is an autonomous tubulin detyrosinase. (A) Sequence homology between LmVASH, TbVASH and HsVASH2. NLS: nuclear localization signal. (B) Identification of nuclear localization signals in human VASH2, *T. brucei* VASH and *L. mexicana* VASH sequences using an NLS mapper (nls-mapper.iab.keio.ac.jp). (C) Immunoblot and (D) immunofluorescence analysis of HEK293 cells showing LmVASH detyrosinase activity in cells expressing the HA-tagged wild-type but not in cells expressing the enzymatically dead (C92A) LmVASH. Scale bar: 5 µm. (E-F) Coomassie gels of 1 µg of the recombinant wild-type or enzymatically dead (C92A) *L. mexicana* VASH (E) and 1 µg of tubulin purified from *Spodoptera frugiperda*-derived Sf9 cells (F). (G) *In vitro* detyrosination assay using the recombinant wild-type or enzymatically dead (C92A) LmVASH. The assay was performed on microtubules polymerized from Sf9-purified tubulin. Reactions were stopped after 30 min and analyzed by immunoblotting with the indicated antibodies. (H) Representative immunoblot of LmVASH activity on microtubules and soluble tubulin from Sf9 insect cells. The assay has been performed three times independently and relative optical density was measured for graphical representation. (I) *In vitro* time course analysis of the recombinant wild-type LmVASH showing specific α-tubulin detyrosination on microtubules. A comparison of LmVASH detyrosinase activity on polymerized (MT) and soluble tubulin purified from Sf9 cells was performed. Samples were analyzed by immunoblotting and relative optical density of three independent assays was measured (n = 3; error bars represent SEM). For soluble Sf9 tubulin, LmVASH activity was below the detection level. The αΔ1 and YL1/2 antibodies recognize detyrosinated and tyrosinated α-tubulin, respectively.

Fig. S2.

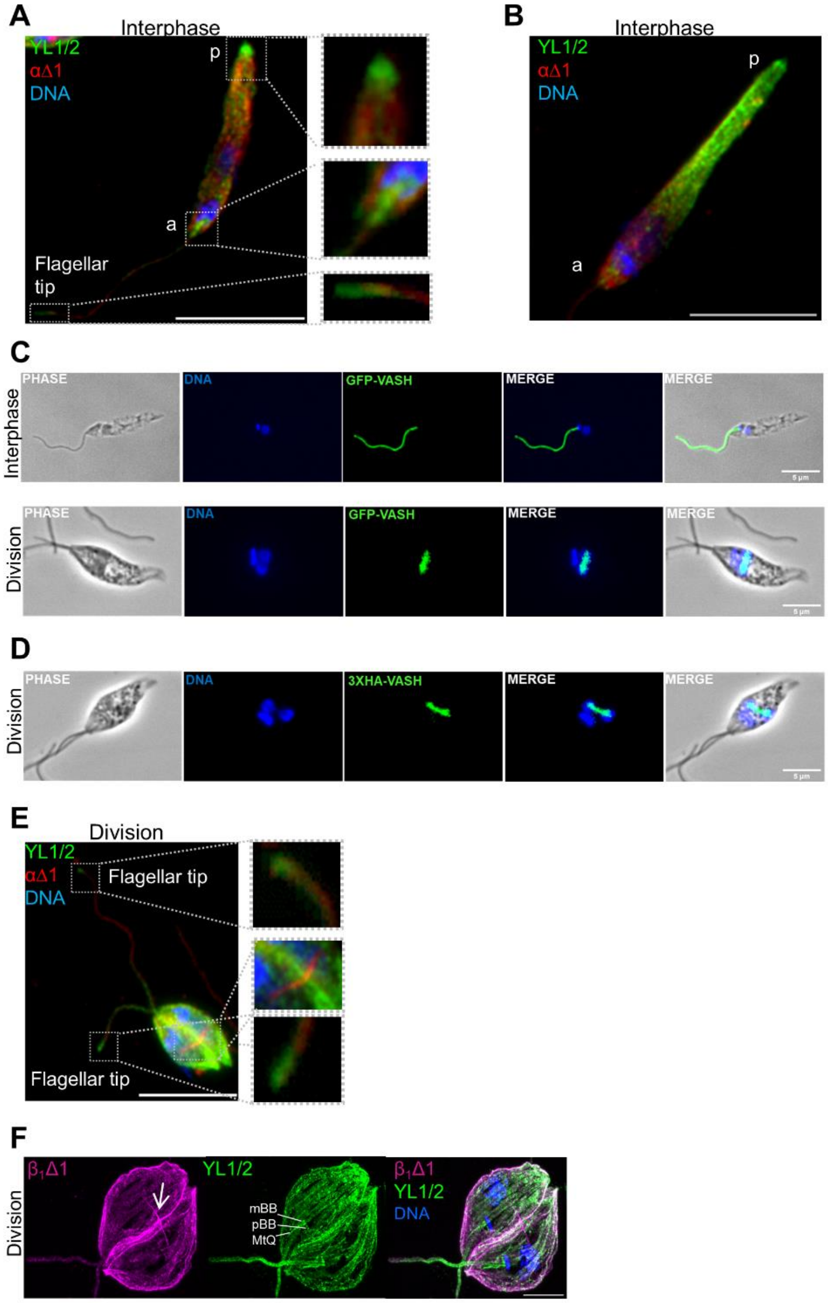
A
Interphase
YL1/2
αΔ1
DNA
p
a
Flagellar tip
B
Interphase
YL1/2
αΔ1
DNA
p
a
C
Interphase
PHASE
DNA
GFP-VASH
MERGE
MERGE
Division
PHASE
DNA
GFP-VASH
MERGE
MERGE
D
Division
PHASE
DNA
3XHA-VASH
MERGE
MERGE
E
Division
YL1/2
αΔ1
DNA
Flagellar tip
Flagellar tip
F
Division
β1Δ1
YL1/2
mBB
pBB
MtQ
β1Δ1
YL1/2
DNA

G

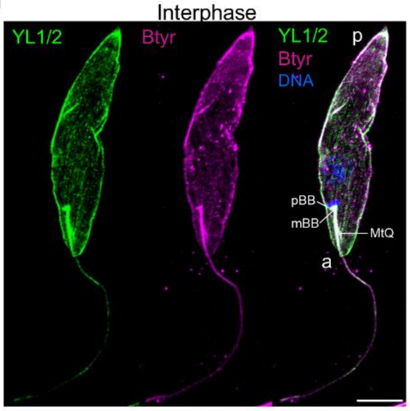

Distinct distribution of α- and β-tubulin detyrosination in *Leishmania* (related to Figure 1). (A-B) Immunofluorescent analysis of tyrosinated and detyrosinated α-tubulin in interphasic *L. mexicana* parental cells. Note the difference between the broad labeling of the subpellicular microtubules in the posterior region of an elongated cell (B), as compared to a shorter cell (A), indicating active microtubule polymerization in this growing region of the cell body. Scale bar: 10 µm. (C) Episomal expression of GFP-LmVASH in parental promastigotes. Scale bar: 5 µm. (D) Immunofluorescent analysis of HA-LmVASH in a dividing parental promastigote Scale bar: 5 µm. (E) Immunofluorescent analysis of tyrosinated and detyrosinated α tubulin in dividing cells from *L. mexicana* parental promastigotes. Scale bar: 10 µm. (F-G) Maximum intensity projections of z-stack confocal images from UExM analysis of tyrosinated α-tubulin and detyrosinated β-tubulin (F) and tyrosinated α and β-tubulin (G) in *L. mexicana* parental promastigotes. (F) The white arrow indicates the mitotic spindle. Scale bar: 10 µm. The YL1/2 antibody recognizes tyrosinated α-tubulin. The αΔ1 antibody recognizes detyrosinated α-tubulin. The β1Δ1 antibody recognizes detyrosinated β-tubulin. The βtyr antibody recognizes tyrosinated β-tubulin. p: posterior; a: anterior; MtQ: microtubule quartet; mBB: mature basal body: pBB: pro basal body.

Fig. S3.

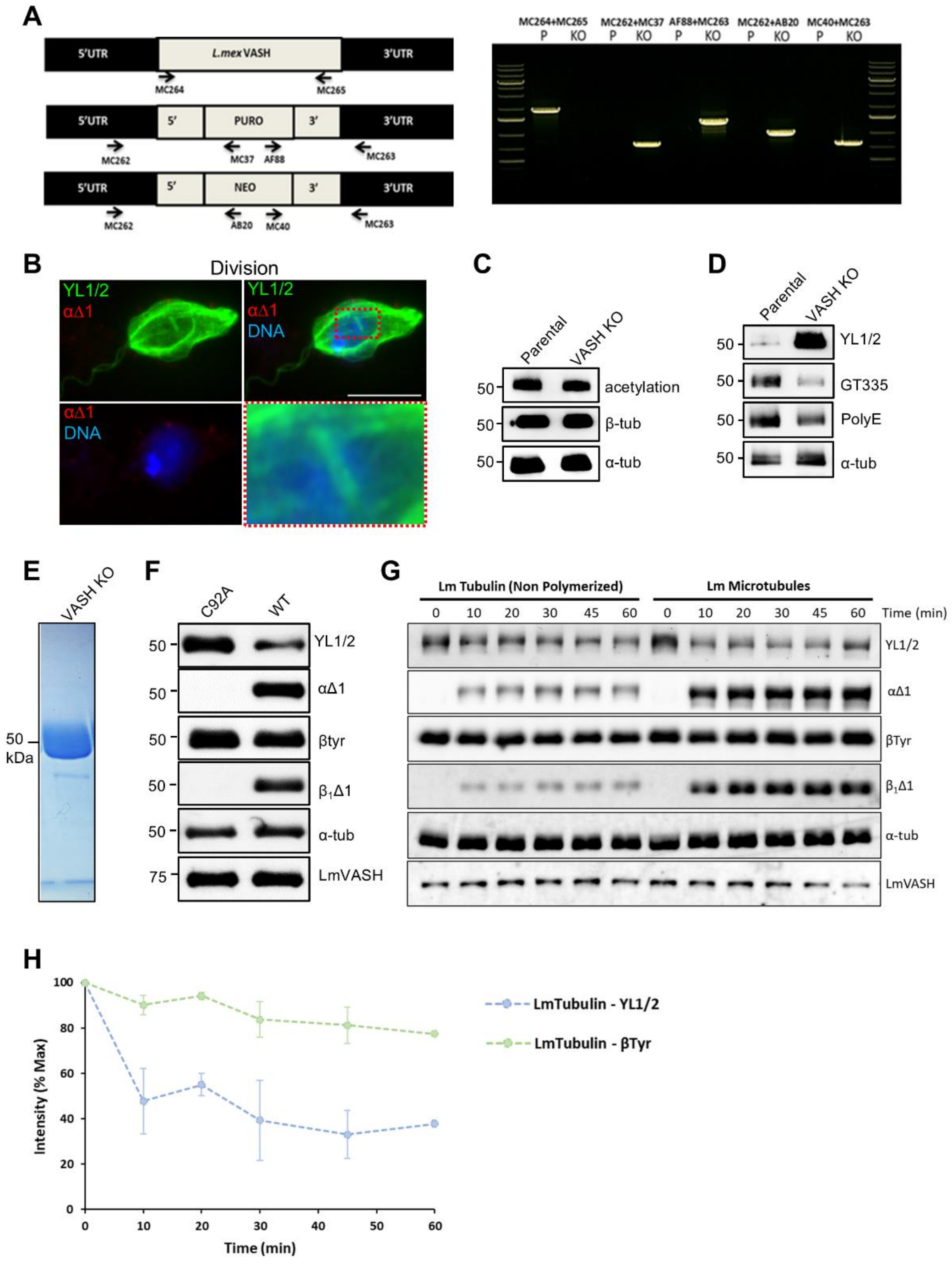

Deletion of LmVASH reveals differential kinetics of detyrosination and a cross-talk with polyglutamylation (related to Figure 2). (A) Diagnostic PCR of LmVASH null mutant promastigotes. Schematic representation of LmVASH gene locus and primers (black arrows) used to confirm integration of the drug-resistant markers and loss of wild-type allele in the LmVASH knockout (KO) promastigotes. Parental cell line (P), Puromycin (PURO), Neomycin (NEO). (B) Immunofluorescent analysis of tyrosinated and detyrosinated α tubulin in dividing cells from LmVASH knockout promastigotes. The αΔ1 antibody recognizes detyrosinated α-tubulin. The

YL1/2 antibody recognizes tyrosinated α-tubulin. Scale bar: 10 µm. (C) Immunoblot analysis of acetylation in *L. mexicana* parental and VASH knockout promastigotes with anti-acetyl-alpha tubulin antibody. (D) Immunoblot analysis of *T. brucei* parental and VASH knockout procyclic cells. The GT335 antibody recognizes glutamyl side chains of any length. The PolyE antibody recognizes polyglutamylation with at least four glutamate residues. The YL1/2 antibody recognizes tyrosinated α-tubulin. (E) Coomassie gel of *L. mexicana* tubulin purified from LmVASH knockout promastigotes after size exclusion chromatography. (F) In vitro detyrosination assay using the recombinant wild-type or enzymatically dead (C92A) LmVASH. The assay was performed on microtubule polymerized from LmVASH knockout promastigotes-purified tubulin. Reactions were stopped after 30 min and analyzed by immunoblotting with the indicated antibodies. Note that only the wild-type enzyme exhibited autonomous activity on both α- and β- polymerized tubulin. (G) Representative immunoblot of LmVASH activity on unpolymerized and polymerized tubulin purified from LmVASH knockout promastigotes. The assay has been performed three times independently and relative optical density was measured for graphical representation as in main Figure 3D. (H) Comparison of detyrosination activity on free tubulin purified from LmVASH knockout promastigotes was performed based on relative optical densities generated by the YL1/2 and the βtyr antibodies.

Fig. S4.

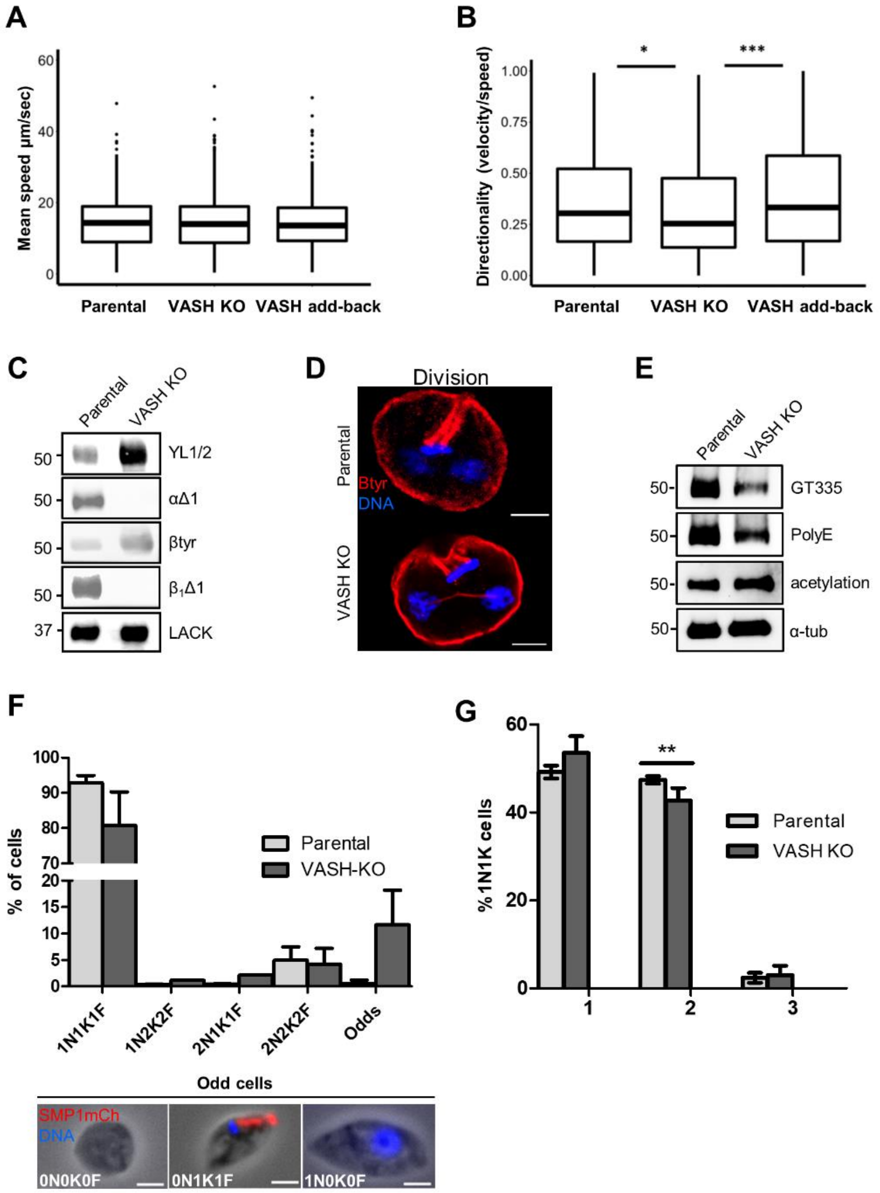

Removal of LmVASH carboxypeptidase activity reduces replication within macrophages and pathogenicity in mice (related to Figure 3). (A) Swimming speed measurements of parental, LmVASH knockout and add-back promastigotes cell lines were calculated from swimming tracks obtained from video microscopy using the plugin Trackmate. (B) Directionality (velocity/speed) measurements of parental, LmVASH knockout and wild-type LmVASH add-back cell lines promastigotes were calculated from swimming tracks obtained from video microscopy using the plugin Trackmate. Boxes and error bars indicate the mean, upper and lower quartiles and 95th percentiles. (C, E) Immunoblot analysis of tubulin PTMs in *L. mexicana* parental and LmVASH knockout amastigotes. (C) The YL1/2 antibody recognizes tyrosinated α-tubulin. The αΔ1 antibody recognizes detyrosinated α-tubulin. The βtyr antibody recognizes tyrosinated β-tubulin. The $\beta_1\Delta1$ antibody recognizes detyrosinated β-tubulin. (E) The GT335 antibody recognizes glutamate side chains of any length. The PolyE antibody recognises polyglutamylation with at least four glutamate residues. (D) UExM analysis of tyrosinated β-tubulin in *L. mexicana* parental and LmVASH

knockout axenic amastigotes cultured over 7 days after differentiation. Scale bar: 5 µm. (F) Quantification of Nuclei (N), kinetoplasts (K) and flagella (F) in *L. mexicana* parental and LmVASH knockout axenic amastigotes cultured over 3 days after differentiation. Microphotographs are representative of the odd cells in the LmVASH knockout axenic amastigotes. (n = 2; error bars represent SD). Scale bar: 1 µm. (G) Fluorescence in situ hybridization (FISH) quantification of parental and LmVASH knockout axenic amastigotes using a chromosome 2-specific probe. Bars indicated mean (n = 3; error bars represent SD). The p values were calculated using t test analysis (n = 3, ** < 0.01).

Fig. S5.

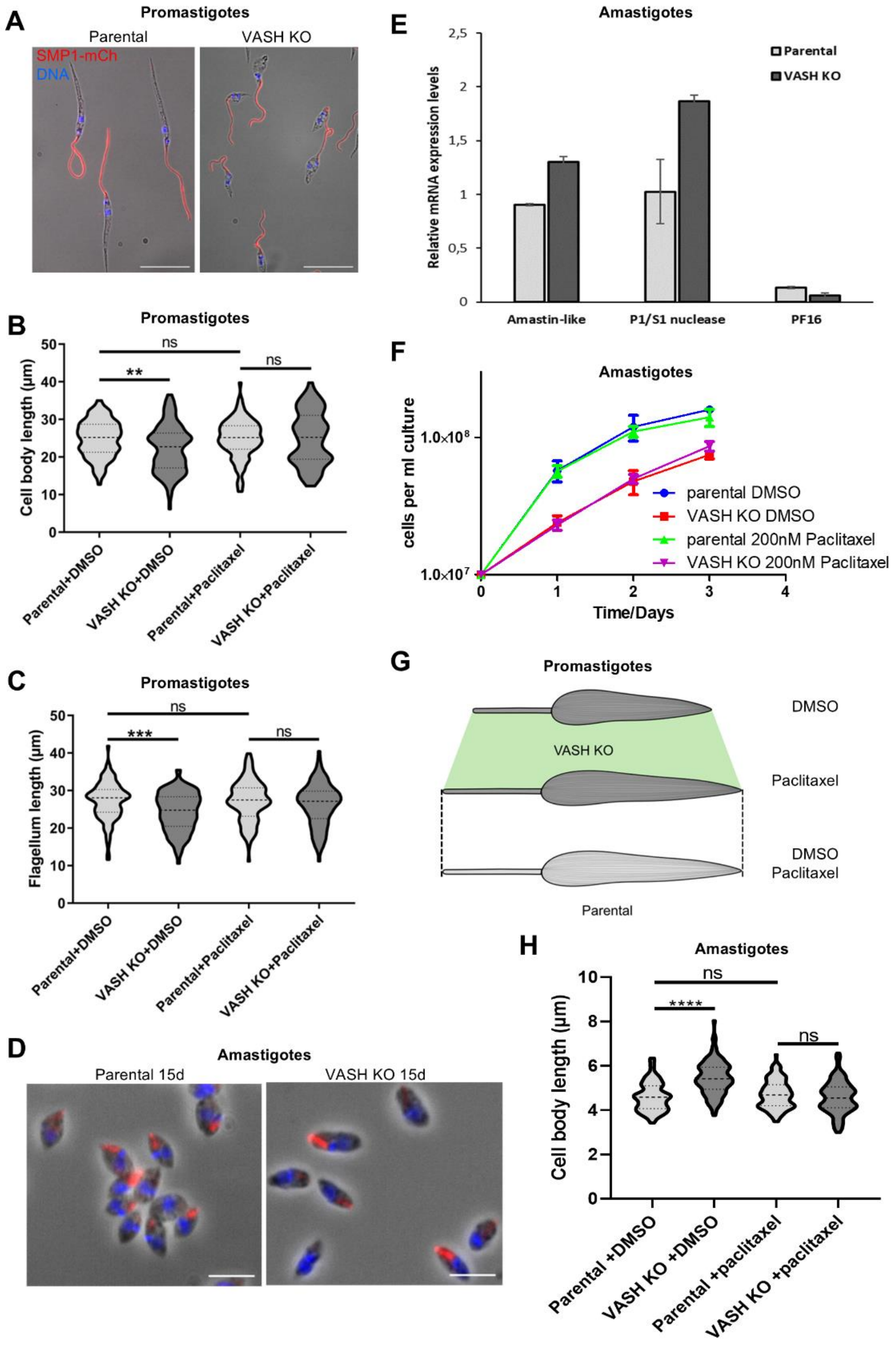

A
Promastigotes
Parental
VASH KO
SMP1-mCh
DNA
B
Promastigotes
Cell body length (µm)
ns
**
ns
Parental+DMSO
VASH KO+DMSO
Parental+Paclitaxel
VASH KO+Paclitaxel
C
Promastigotes
Flagellum length (µm)
ns
***
ns
Parental+DMSO
VASH KO+DMSO
Parental+Paclitaxel
VASH KO+Paclitaxel
D
Amastigotes
Parental 15d
VASH KO 15d
E
Amastigotes
Relative mRNA expression levels
Parental
VASH KO
Amastin-like
P1/S1 nuclease
PF16
F
Amastigotes
cells per ml culture
parental DMSO
VASH KO DMSO
parental 200nM Paclitaxel
VASH KO 200nM Paclitaxel
Time/Days
G
Promastigotes
DMSO
VASH KO
Paclitaxel
DMSO
Paclitaxel
Parental
H
Amastigotes
Cell body length (µm)
ns
****
ns
Parental +DMSO
VASH KO +DMSO
Parental +paclitaxel
VASH KO +paclitaxel

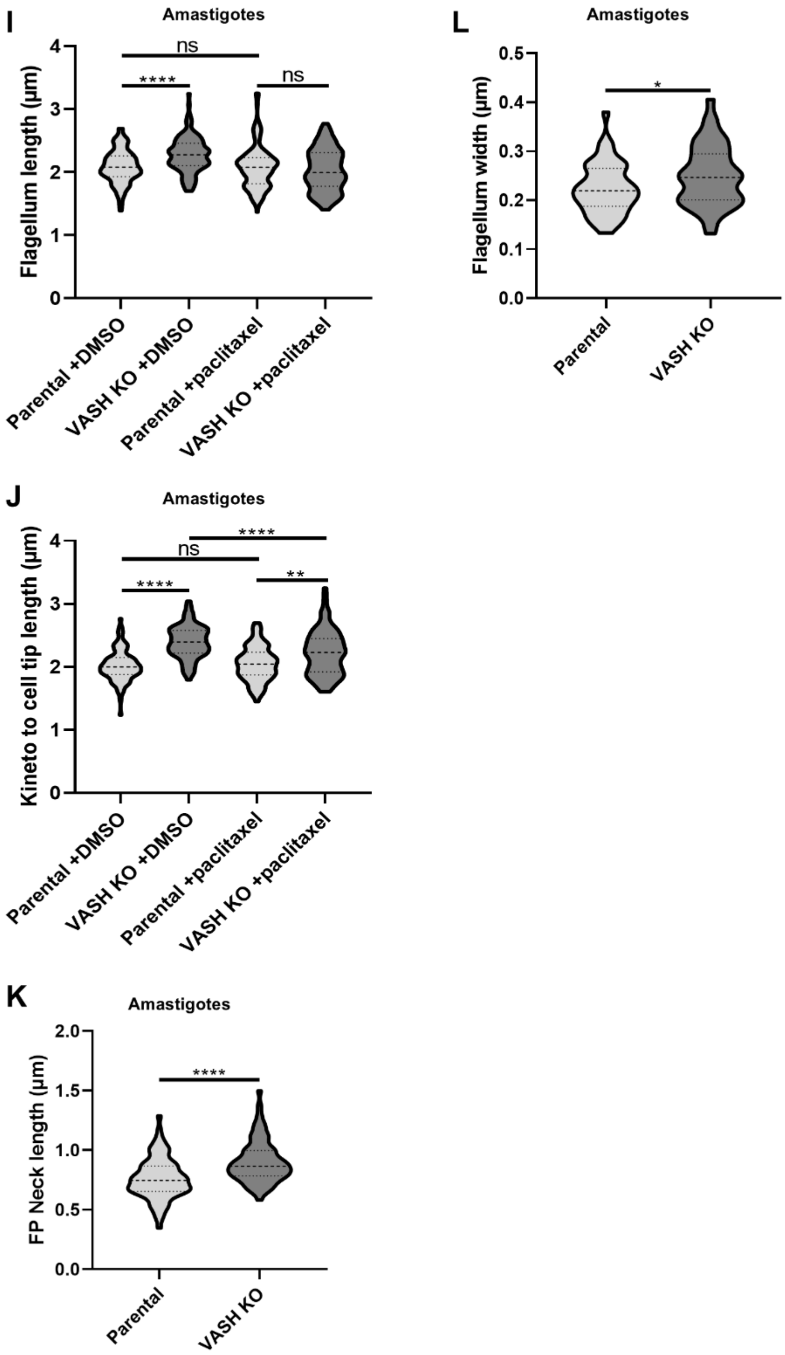

Lack of detyrosination impacts morphogenesis and flagellar pocket shape (related to Figure 4). (A) Fluorescence micrographs of stationary promastigotes from parental and LmVASH knockout cells expressing the flagellar membrane-associated SMP1 fused with mCherry. Note the shorter flagellum and cell body in LmVASH knockout cells. Scale bar: 20 µm. (B) Cell body and (C) flagellum length measurements of stationary promastigotes from parental and LmVASH knockout cells treated during 24h with DMSO (control) or 200nM paclitaxel. Cells were fixed with PFA at a density of 2 x10^7 cells/ml. Fifty cells were counted per sample (n=3). Dotted lines indicate the median, upper and lower quartiles. **p < 0.01, ***p < 0.001 (Anova-one way). (D) Fluorescence micrographs of axenic amastigotes cultured over 15 days after differentiation from parental and LmVASH knockout cells expressing SMP1 fused with mCherry. Scale bar: 5 µm. (E) Quantitative

PCR (qPCR) of amastigotes (Amastin-like and P1/S1 nuclease) and promastigotes (PF16) molecular markers performed on *L. mexicana* axenic amastigotes from parental and LmVASH knockout cells cultured after 7 days of differentiation. (F) Growth curve (log scale) of *L. mexicana* axenic amastigotes from parental and LmVASH knockout cells cultured after 6 days of differentiation and treated during 3 days with 200nM paclitaxel. Cell density was determined by counting at 24 h intervals (n = 3; error bars represent SD). (G) Cartoon summarizing the effects of dynamic instability in the cell morphology of promastigotes from LmVASH knockout cells. Paclitaxel-induced microtubule stability restores LmVASH knockout morphological phenotypes with neutral effects on the parental cell line. Not to scale. (H) cell body, (I) flagellum length and (J) kinetoplast to cell body end measurements of axenic amastigotes from parental and LmVASH knockout cells cultured after 3 days of differentiation and treated during 4 days with a single dose of 200nM paclitaxel. A total of 100 cells from two independent experiments were counted for each cell line. Dotted lines indicate the median, upper and lower quartiles. $^{**}p < 0.01$ $^{****}p < 0.0001$ (Anova-one way). (K) Flagellar pocket neck length measurements of axenic amastigotes from parental and LmVASH knockout cells. A total of 75 longitudinal sections through the flagellar pocket from parental and LmVASH knockout cells were counted. Dotted lines indicate the median, upper and lower quartiles. $^{****}p < 0.0001$ (Mann-Whitney test). (L) Flagellum width measurements (parental: n=44 and LmVASH knockout: n=40) of axenic amastigotes at the constriction point at the distal end of the flagellar pocket neck. Dotted lines indicate the median, upper and lower quartiles. $^{*}p < 0.05$ (unpaired Student's t-test t).

Fig. S6.

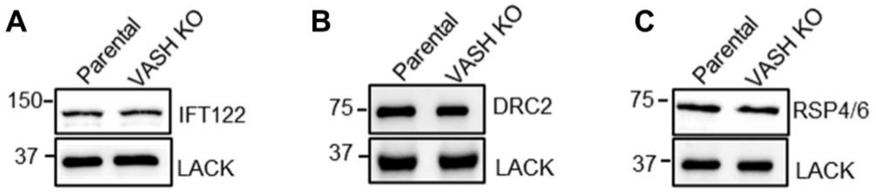

Tubulin detyrosination promotes the remodeling of the amastigote flagellum_(related to Figure 5). (A, B, C) Western blot of detergent-extracted cytoskeletons from the parental and LmVASH knockout axenic amastigotes expressing (A) IFT122-3XHA, (B) DRC2-3XHA or (C) RSP4/6-3XHA. $5x10^7$ cytoskeletons were loaded on a 10% SDS-PAGE, transferred and immuno-probed with anti-HA and anti-LACK (loading control) antibodies.

Fig. S7.

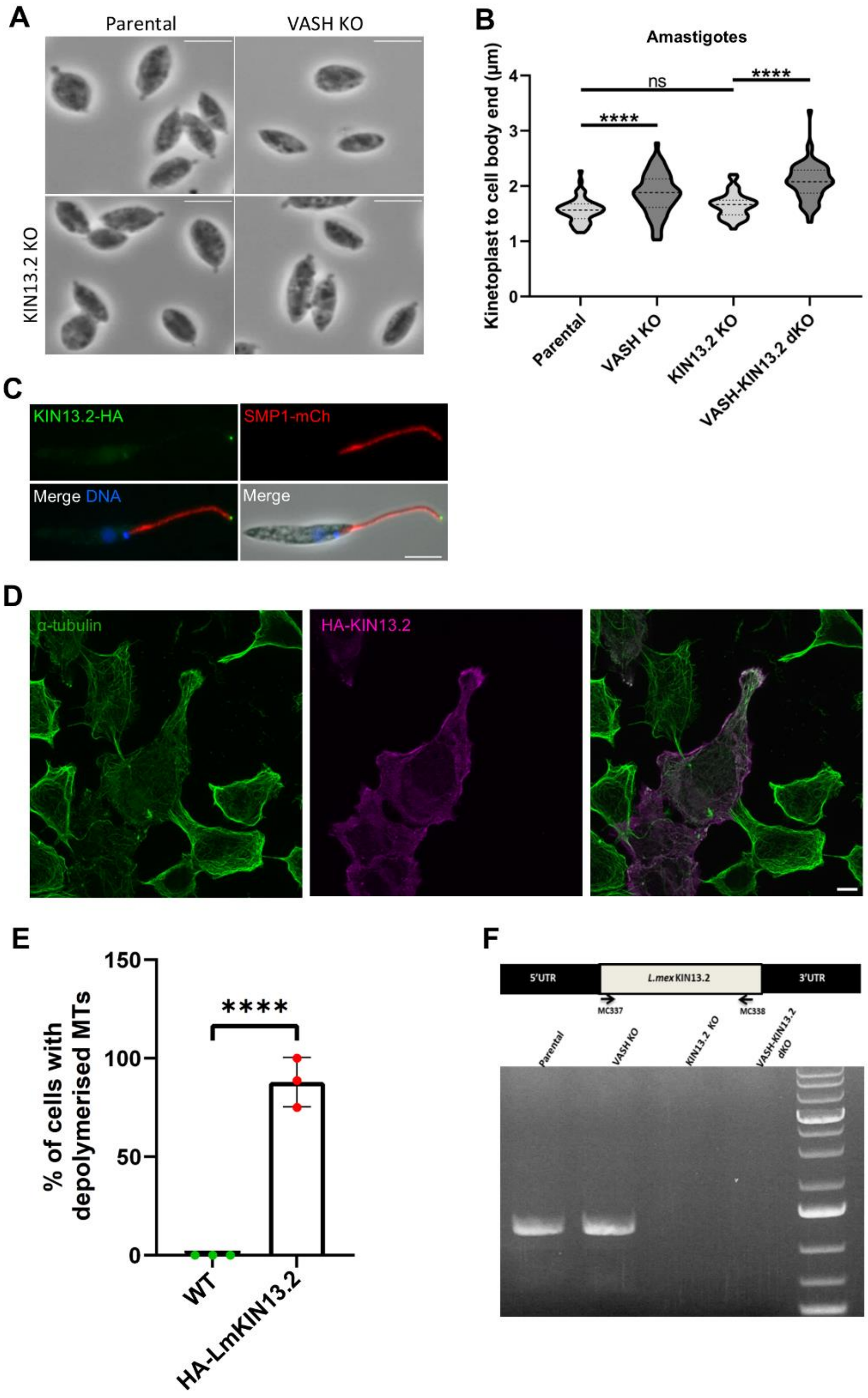

Microtubule depolymerizing activity of the flagellar LmKIN13.2 is increased in the absence of detyrosination (related to Figure 6). (A) Bright-field micrographs of axenic amastigotes from parental, LmVASH knockout, LmKIN13.2 knockout and LmVASH-LmKIN13.2 double knockout cell lines cultured over 3 days after differentiation. Scale bar: 5 µm. (B) Kinetoplast to cell body end measurements of axenic amastigotes from parental, LmVASH knockout, LmKIN13.2 knockout and LmVASH-LmKIN13.2 double knockout cell lines cultured over 3 days after differentiation and

expressing SMP1 fused with mCherry. Cells were fixed with PFA at a density of $3x10^7$ cells/ml. Fifty cells were counted per sample (n=3). Dotted lines indicate the median, upper and lower quartiles. ****p < 0.0001 (Anova-one way). (C) Immunofluorescent analysis of axenic promastigotes co-expressing HA-tagged LmKin13-2 and the flagellar membrane-associated SMP1 fused with mCherry. Cells were probed with anti-mCherry and anti-HA antibodies. Scale bar: 5µm. (D) Microtubule-depolymerizing activity of the *L. mexicana* Kinesin-13.2. Immunofluorescent analysis of tubulin in U2OS cells expressing LmKIN13.2-HA. Scale bar: 10 µm. (E) Pourcentage of the number of RPE1 cells with depolymerized microtubules (MTs). 264 wild-type cells and 340 cells expressing HA-LmKIN13.2 were counted. Non-parametric t-test (Mann-Whitney), p<0.0001. (F) Diagnostic PCR of LmKIN13.2 null mutant promastigotes. Schematic representation of LmKIN13.2 gene locus and primers (black arrows) used to confirm loss of wild-type allele in the parental and LmVASH knockout (KO) promastigotes.

Table S1. Primers used for DNA constructs.

Primers	5' to 3' Sequence	Target
LmVASH_Fw (MfeI)	GGATTATGCTCAATTGATGTCGGATAAAGTGCTGGCCA	LmVASH ORF
LmVASH_Rv (HpaI)	ATGGTGATGGTGGTTAACCTACCGGCGTGGTGAACACA	LmVASH ORF
LmVASH_Fw (BamHI)	AAATATGGATCCATGTCGGATAAAGTGCTGGCC	LmVASH ORF
LmVASH_Rv (EcoRI)	TAAATTGAATTCCTACCGGCGTGGTGAGCACAT	LmVASH ORF
LmVASH VASH_C92A _Fw	CATCCGCGCCCTCGAGGCAACCTTTGTGGC	LmVASH ORF
LmVASH VASH_C92A_Rv	TTGCCTCGAGGGCGCGGATGGGTAGTG	LmVASH ORF
MC264_Fw	ATGTCGGATAAAGTGCTGGC	LmVASH ORF
MC265_Rv	CCGGCGTGGTGAACACATG	LmVASH ORF
MC37_Rv	TCAATGTGTCGATCTGGGTCAAC	PUROMYCIN
AF88_Fw	CCCAGATCGACACATTGAGCG	PUROMYCIN
AB20_Fw	ATCGACAAGACCGGCTTCC	NEOMYCIN
MC40_Rv	ACCGCTTCCTCGTGCTTTA	NEOMYCIN
MC262_Fw	CGTAGCGGCCTGTGTGACC	LmVASH 5'UTR
MC263_Rv	GGCAAGATAGGAGCCGCAC	LmVASH 3'UTR
MC337_Fw	ATGCGAGAGGACAGCCCC	LmKIN13.2 ORF
MC338_Rv	CTTGTCCAGACGCTCCATCG	LmKIN13.2 ORF
LmKIN13.2_Fw	CGCCGCGGATCCATGCGAGAGGACAGCCC	LmKIN13.2 ORF
LmKIN13.2_Rv	CCGGGAATTCTCACTTGTCCAGACGCTCCAT	LmKIN13.2 ORF
LmGPDH_Fw	TACTTCGACAACATTGGCATTAT	LmGPDH ORF
LmGPDH_Rv	AGAACACAATTCTCCTTAGTTACTG	LmGPDH ORF
LmxM.08.0850-AMASTIN_Fw	CATGGTGGTGGTCTACTACA	LmAMASTIN ORF
LmxM.08.0850-AMASTIN_Rv	GATCCGTAGCGTTGAAGGTAA	LmAMASTIN ORF
LmxM.29.1500. P1/S1-NUCLEASE_Fw	GTATGGTGACCTCGCTTAAGAA	LmP1/S1-NUCLEASE ORF
LmxM.29.1500. P1/S1-NUCLEASE_Rv	CGTTGCCTCCTTGATCC	LMP1/S1-NUCLEASE ORF
LmxM.20.1400-PF16_Fw	GAGGTGGTGGACAAAGG	LmPF16 ORF
LmxM.20.1400-PF16_Rv	GCCAGGTGGGTGATAGC	LmPF16 ORF

Movie S1 (separate file). (related to Figure 2). Confocal z-stack images from expansion microscopy of *Leishmania* promastigotes labelled with αΔ1 and YL1/2 antibodies

Movie S2 (separate file). (related to Figure 2). Confocal z-stack images from expansion microscopy of *Leishmania* promastigotes labelled with αΔ1 and YL1/2 antibodies

Movie S3 (separate file). (related to Figure 2). Confocal z-stack images from expansion microscopy of *Leishmania* promastigotes labelled with β1Δ1 and YL1/2 antibodies

Movie S4 (separate file). (related to Figure 2). Confocal z-stack images from expansion microscopy of *Leishmania* promastigotes labelled with βtyr and YL1/2 antibodies

Movie S5 (separate file). (related to Figure 2). Confocal z-stack images from expansion microscopy of *Leishmania* promastigotes labelled with βtyr and YL1/2 antibodies

SI References

1. T. Beneke, *et al.*, A CRISPR Cas9 high-throughput genome editing toolkit for kinetoplastids. *R Soc Open Sci* **4**, 170095 (2017).

2. S. van der Laan, *et al.*, Evolutionary Divergence of Enzymatic Mechanisms for Tubulin Detyrosination. *Cell Rep* **29**, 4159-4171.e6 (2019).

3. C. Halliday, *et al.*, Cellular landmarks of Trypanosoma brucei and Leishmania mexicana. *Mol Biochem Parasitol* **230**, 24–36 (2019).

4. R. M. Corrales, *et al.*, The kinesin of the flagellum attachment zone in Leishmania is required for cell morphogenesis, cell division and virulence in the mammalian host. *PLoS Pathog* **17**, e1009666 (2021).

5. J.-Y. Tinevez, *et al.*, TrackMate: An open and extensible platform for single-particle tracking. *Methods* **115**, 80–90 (2017).

6. Y. Sterkers, L. Lachaud, L. Crobu, P. Bastien, M. Pagès, FISH analysis reveals aneuploidy and continual generation of chromosomal mosaicism in Leishmania major. *Cell Microbiol* **13**, 274–283 (2011).
